# Supplementary material for: Interstellar formation of lactaldehyde, a key intermediate in the methylglyoxal pathway
Source: Nat Commun. 2024 Nov 24;15:10189. doi: 10.1038/s41467-024-54562-x (PMC11586434; doi:10.1038/s41467-024-54562-x)
Supplement: Supplementary file 1 — Supplementary Information [file 41467_2024_54562_MOESM1_ESM.pdf]

Supplementary Information for

Interstellar Formation of Lactaldehyde, A Key Intermediate in the Methylglyoxal Pathway

Jia Wang[1,2], Chaojiang Zhang[1,2], Joshua H. Marks[1,2], Mikhail M. Evseev[3], Oleg V. Kuznetsov[3], Ivan O. Antonov[3*], Ralf I. Kaiser[1,2*]

[1] W. M. Keck Research Laboratory in Astrochemistry, University of Hawaii at Manoa, Honolulu, HI 96822, USA

[2] Department of Chemistry, University of Hawaii at Manoa, Honolulu, HI 96822, USA

[3] Samara National Research University, Samara 443086, Russia

[*]Corresponding Author:

Ivan Antonov, pfizeke@gmail.com

Ralf I. Kaiser, ralfk@hawaii.edu

This file includes:

Assignment of the molecular formula

Isomerization potential energy surfaces of lactaldehyde

Electron irradiation as a proxy for GCR interactions within interstellar ices

Supplementary Figures 1 to 16

Supplementary Tables 1 to 19

Supplementary References

Supplementary Note 1: Assignment of the molecular formula

For the CO–CH_3CH_2OH system, the ion signal at *m/z* = 74 can belong to organic compounds with formulae including C_6H_2, $C_4H_{10}O$, $C_3H_6O_2$, and $C_2H_2O_3$. Recall that the fully isotopic labeling experiments with ^{13}C and D, and the partially ^{18}O isotopic labeled experiment confirm the inclusion of exactly six hydrogen atoms, exactly three carbon atoms, and at least one oxygen atom; hence verifying the molecular formula $C_3H_6O_2$ in CO–CH_3CH_2OH ice. The formulae such as C_3H_6S, $C_2H_6N_2O$, and CH_6N_4 are not possible. Furthermore, other potential formulae can be ruled out (Supplementary Table 19).

Supplementary Note 2: Isomerization potential energy surfaces of lactaldehyde (7)

We explored the isomerization potential energy surfaces of **7** in the gas phase at the composite CBS-QB3 level of theory (Supplementary Fig. 15). Our calculations show that the reactions of forming **21** (reaction [9a]) and **24** (reaction [9b]) are endoergic by 48 kJ mol^{-1} and 18 kJ mol^{-1}, respectively, which are close to the previous calculated results at the G3X-K level of theory[1]. In addition, the reaction [9b] forming **24** has a barrier of 185 kJ mol^{-1}, which is significantly lower than the highest barrier (286 kJ mol^{-1}) of reaction [9a] to **21**; this finding indicates that the formation of **24** is likely preferred. We performed an additional experiment by adding 1% of **24** to the reactants at 11.10 eV under identical experimental conditions, but without radiation exposure. The TPD profile of **24** at *m/z* = 74 shows two sublimation events peaking at 152 K and 175 K (Supplementary Fig. 16); the first peak is due to the co-sublimation with **15**. Since the second sublimation peak (175 K) of **24** is very close to the peak sublimation temperature of **18** (178 K) and the IEs of **24** (IE = 9.87–10.07 eV)[2] and **18** (IE = 9.73–10.10 eV) overlap, the identification of **24** is inconclusive under current experimental conditions.

$CH_3CH(OH)CHO$ (**7**) ⇋ $CH_3C(OH)CHOH$ (**21**) (+ 48 kJ mol^{-1}) [9a]

$CH_3CH(OH)CHO$ (**7**) → $CH_3C(O)CH_2OH$ (**24**) (+ 18 kJ mol^{-1}) [9b]

Supplementary Note 3: Electron irradiation as a proxy for GCR interactions within interstellar ices

The current simulation experiments were performed to unravel the formation pathways of complex organic molecules in interstellar ices in cold molecular clouds through the interaction with GCRs. The main constituents of GCRs are energetic protons (H^+) and helium nuclei (He^{2+})[3]. It is important to note that no laboratory experiment can directly mimic the interaction of energetic GCRs with ices due to the lack of experimental device that can generate a broad range (from MeV to the PeV) of kinetic energies of protons and helium nuclei[4]. However, the physical effects of GCRs interacting with ices are known. GCRs primarily lose energy through ionization of the target molecules in the ice and generate secondary electrons that can induce further ionization, resulting in electron cascades[5]. Consequently, the kinetic energy of the resulting electrons are in ranges of a few eV up to 10 keV depending on the energy of the GCR particle[6,7]. These electrons, especially for low-energy (< 20 eV) secondary electrons, could be a significant contributor to the interstellar synthesis of prebiotic molecules[7-9]. Therefore, the chemical effects of GCRs on ices can be simulated by irradiating the ices with energetic electrons as a proxy[10,11]. The present experiments utilized the electron kinetic energies of 5 keV as their linear energy transfer is similar to that of 10–20 MeV GCR protons deposit into ices[12,13]. In addition, 5 keV electrons have been widely used previously to simulate the secondary electrons released during GCRs penetrating interstellar ices[10,14-16].

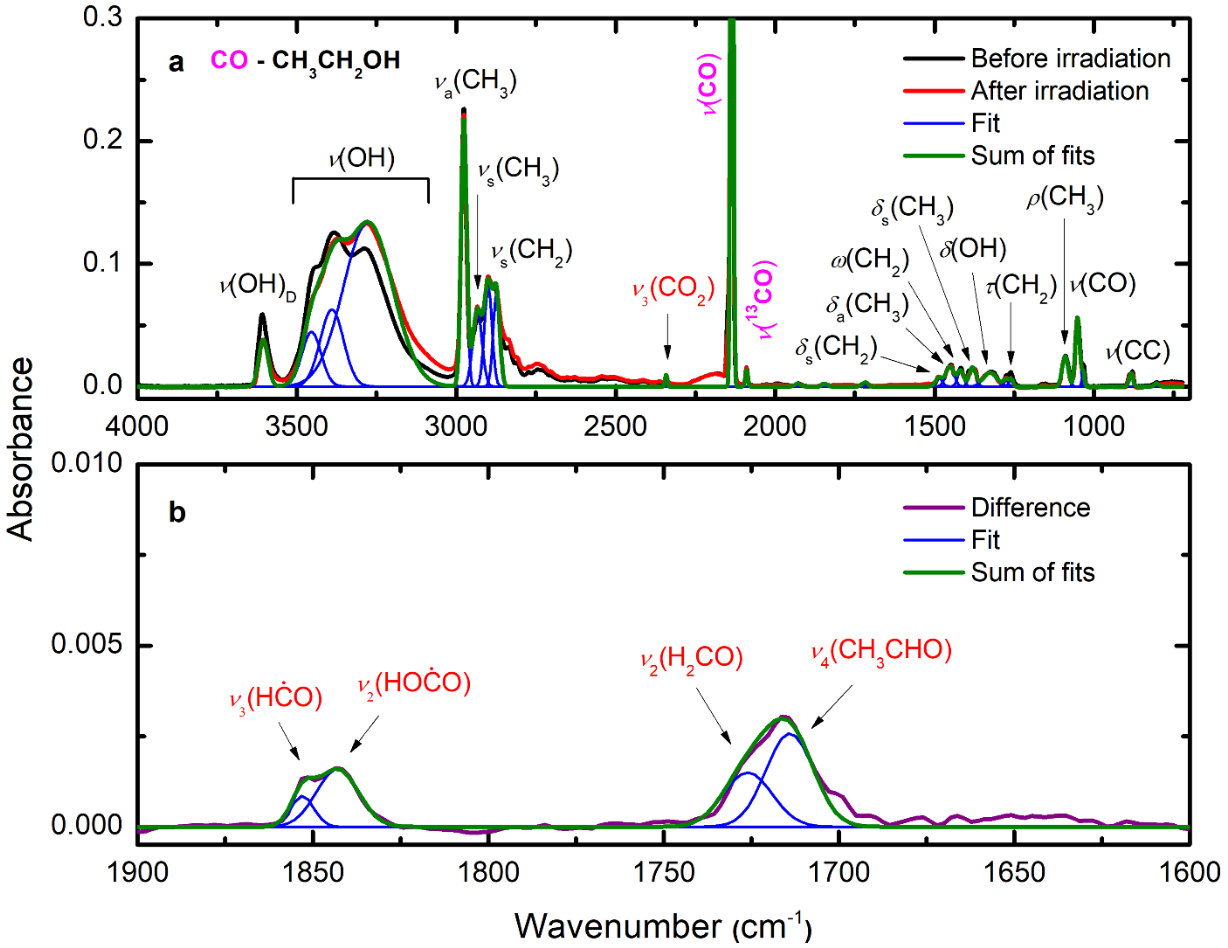

Supplementary Figure 1. Infrared spectra of CO−CH_3CH_2OH ice with low dose irradiation. Infrared spectra of (**a**) CO−CH_3CH_2OH ice before (black) and after (red) low dose irradiation (23 nA, 5 minutes) at 5 K with (**b**) a magnified view and deconvolution (Gaussian) of the region 1900–1600 cm^{-1} of the difference spectrum. The assignments of the absorptions of CO, CH_3CH_2OH, and new absorptions after irradiation are labeled in magenta, black, and red, respectively. Detailed assignments are compiled in Supplementary Table 1.

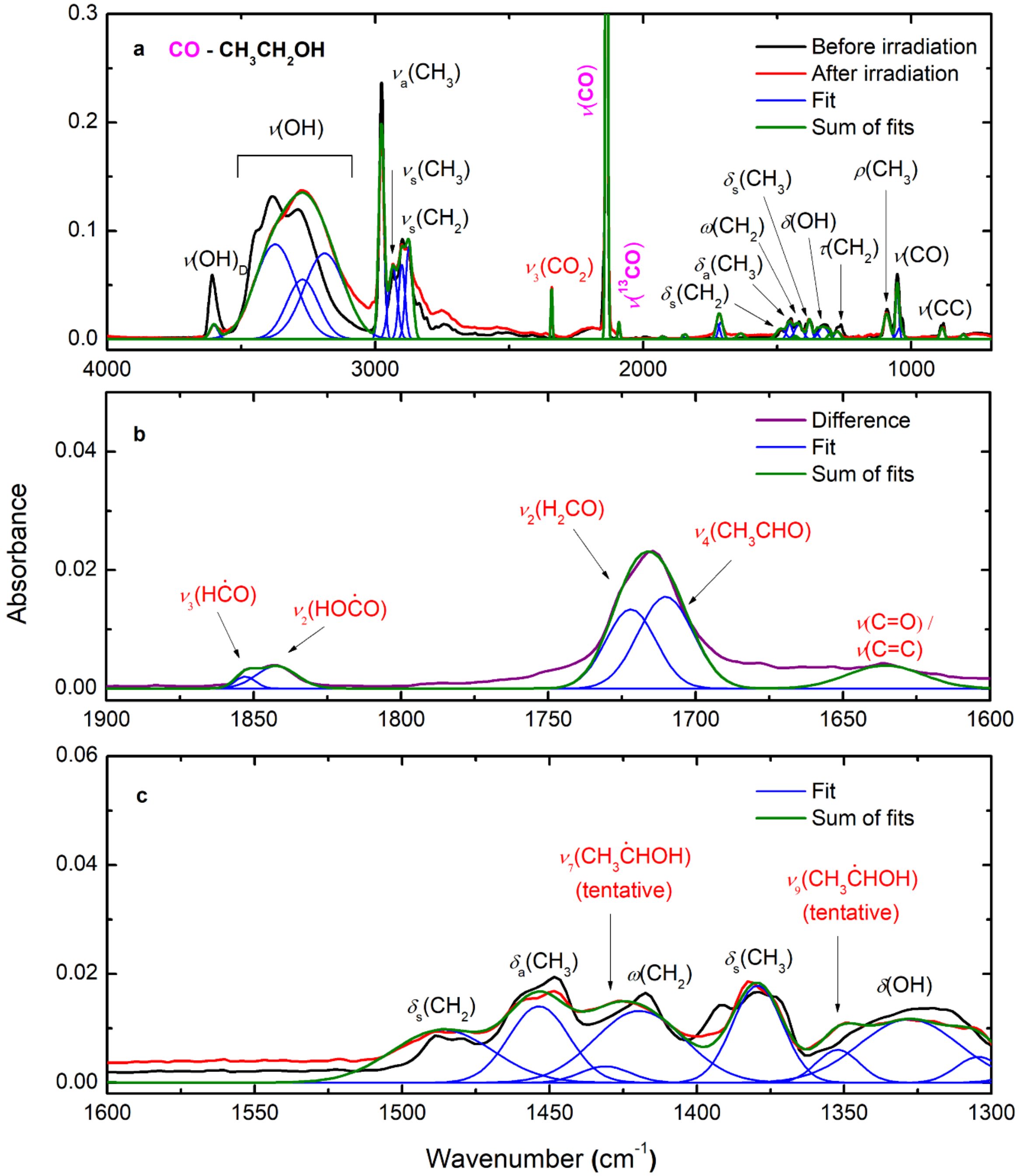

Supplementary Figure 2. Infrared spectra of CO−CH_3CH_2OH ice with high dose irradiation. Infrared spectra of (**a**) CO−CH_3CH_2OH ice before (black) and after (red) high dose irradiation (123 nA, 10 minutes) at 5 K with the magnified view and deconvolution (Gaussian) of the regions (**b**) 1900–1600 cm^{-1} and (**c**) 1600–1300 cm^{-1}. The assignments of the absorptions of CO, CH_3CH_2OH, and new absorptions after irradiation are labeled in magenta, black, and red, respectively. Detailed assignments are compiled in Supplementary Table 2.

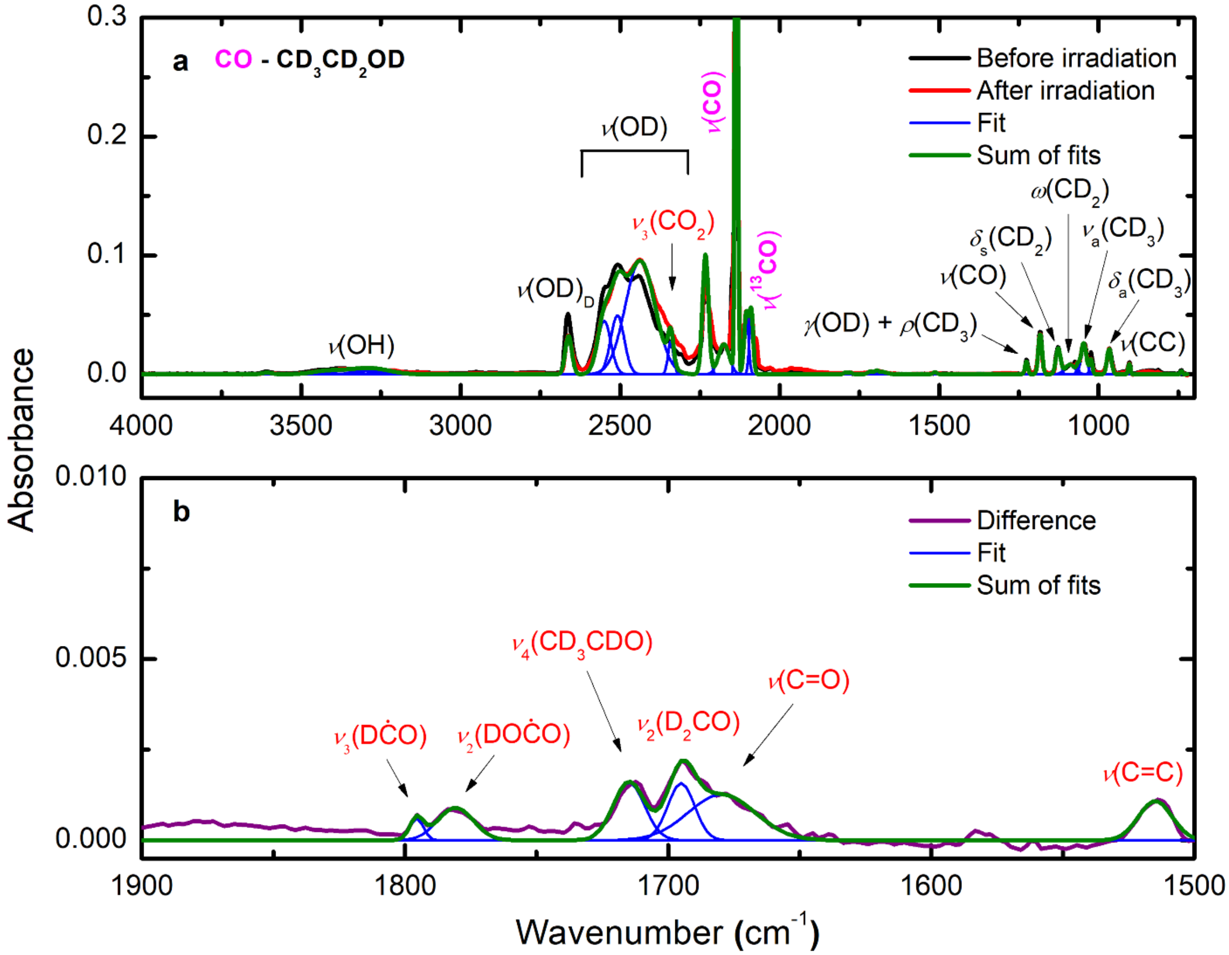

Supplementary Figure 3. Infrared spectra of CO−CD_3CD_2OD ice with low dose irradiation. Infrared spectra of (**a**) CO−CD_3CD_2OD ice before (black) and after (red) low dose irradiation (23 nA, 5 minutes) at 5 K with (**b**) a magnified view and deconvolution (Gaussian) of the region 1900–1500 cm^{-1} of the difference spectrum. The assignments of the absorptions of CO, CD_3CD_2OD, and new absorptions after irradiation are labeled in magenta, black, and red, respectively. Detailed assignments are compiled in Supplementary Table 3.

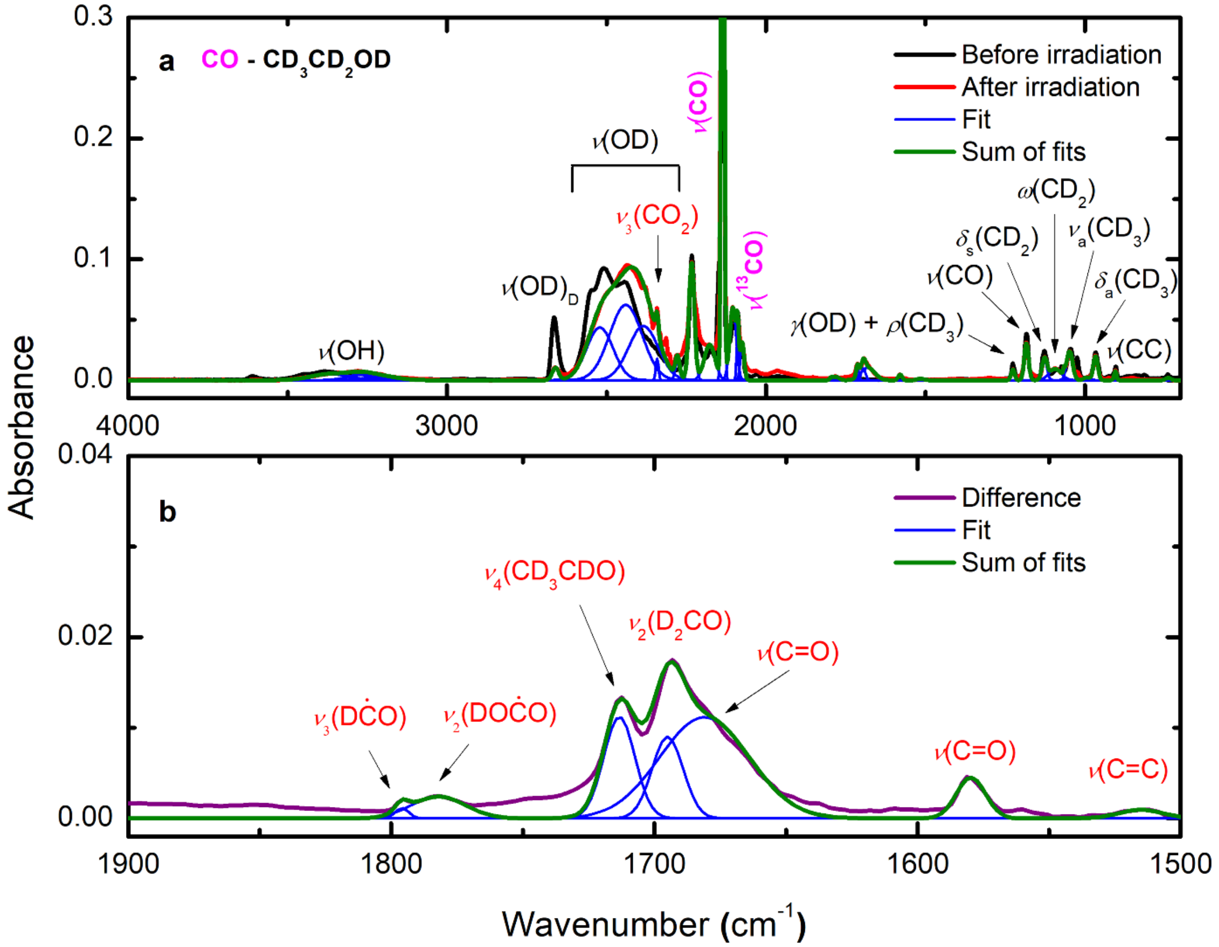

Supplementary Figure 4. **Infrared spectra of CO−CD_3CD_2OD ice with high dose irradiation.** Infrared spectra of (**a**) CO−CD_3CD_2OD ice before (black) and after (red) high dose irradiation (123 nA, 10 minutes) at 5 K with (**b**) a magnified view and deconvolution (Gaussian) of the region 1900–1500 cm^{-1} of the difference spectrum. The assignments of the absorptions of CO, CD_3CD_2OD, and new absorptions after irradiation are labeled in magenta, black, and red, respectively. Detailed assignments are compiled in Supplementary Table 4.

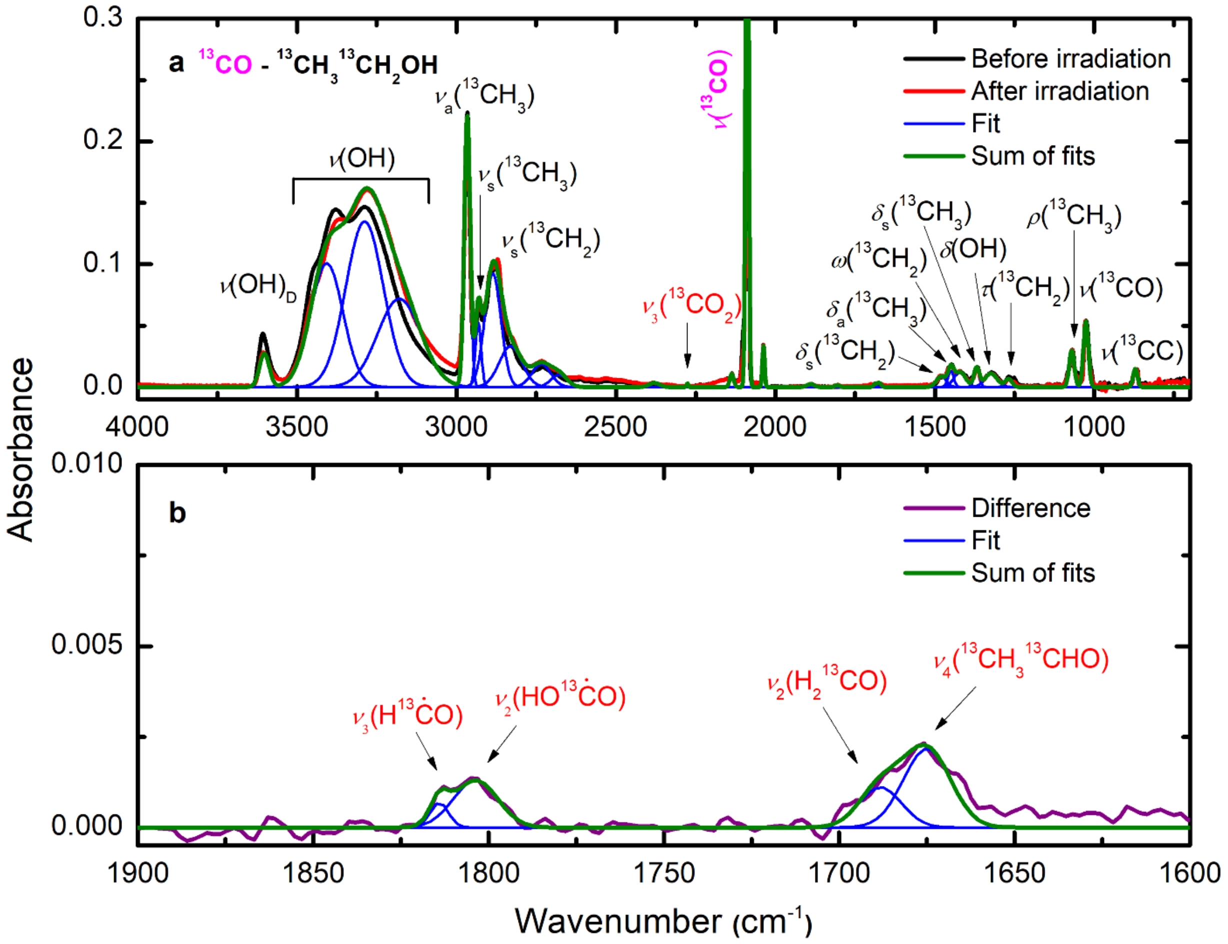

Supplementary Figure 5. Infrared spectra of ^{13}CO–$^{13}CH_3{}^{13}CH_2OH$ ice with low dose irradiation. Infrared spectra of (**a**) ^{13}CO–$^{13}CH_3{}^{13}CH_2OH$ ice before (black) and after (red) low dose irradiation (22 nA, 5 minutes) at 5 K with (**b**) a magnified view and deconvolution (Gaussian) of the region 1900–1600 cm^{-1} of the difference spectrum. The assignments of the absorptions of ^{13}CO, $^{13}CH_3{}^{13}CH_2OH$, and new absorptions after irradiation are labeled in magenta, black, and red, respectively. Detailed assignments are compiled in Supplementary Table 5.

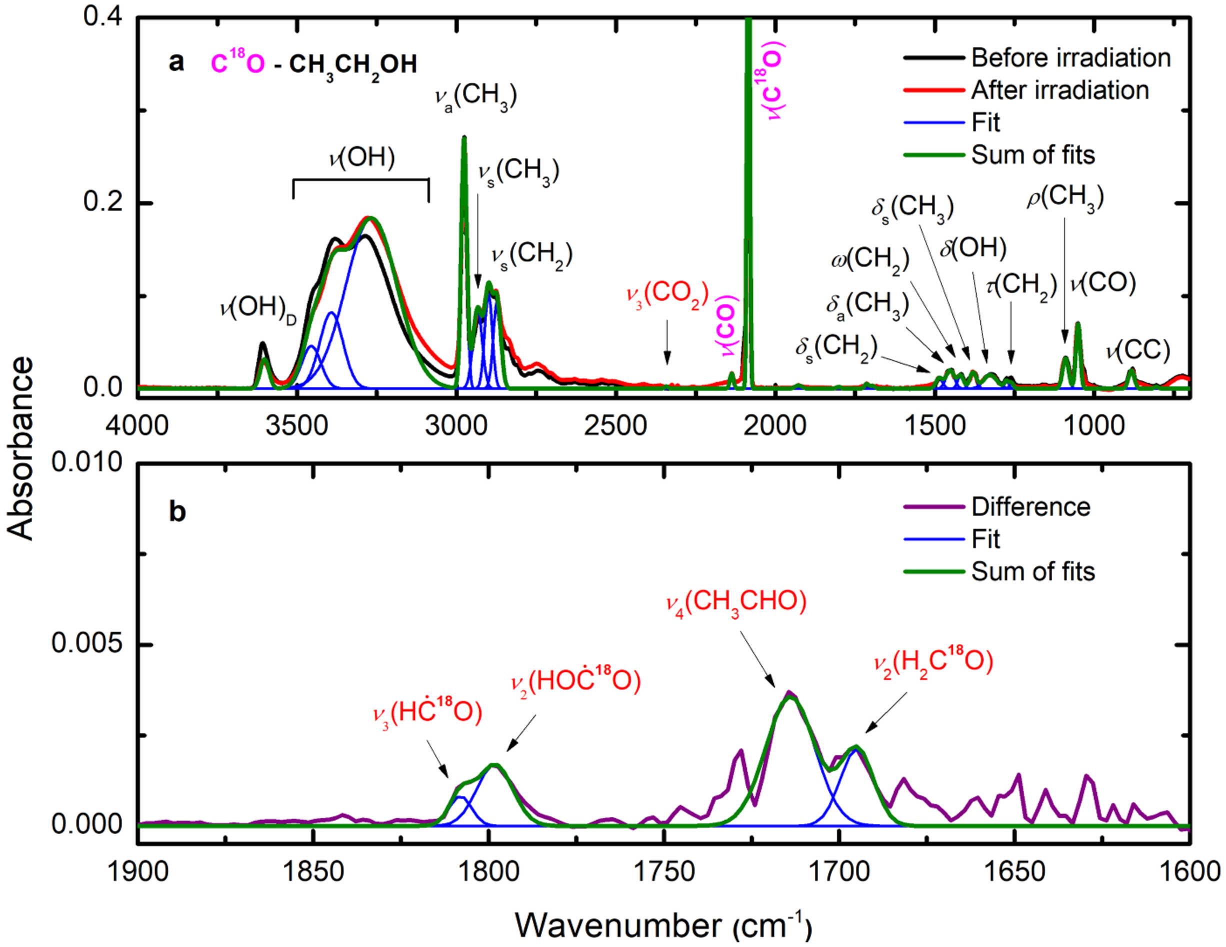

Supplementary Figure 6. Infrared spectra of $C^{18}O$−CH_3CH_2OH ice with low dose irradiation. Infrared spectra of (**a**) $C^{18}O$−CH_3CH_2OH ice before (black) and after (red) low dose irradiation (24 nA, 5 minutes) at 5 K with (**b**) a magnified view and deconvolution (Gaussian) of the region 1900–1600 cm^{-1} of the difference spectrum. The assignments of the absorptions of $C^{18}O$, CH_3CH_2OH, and new absorptions after irradiation are labeled in magenta, black, and red, respectively. Detailed assignments are compiled in Supplementary Table 6.

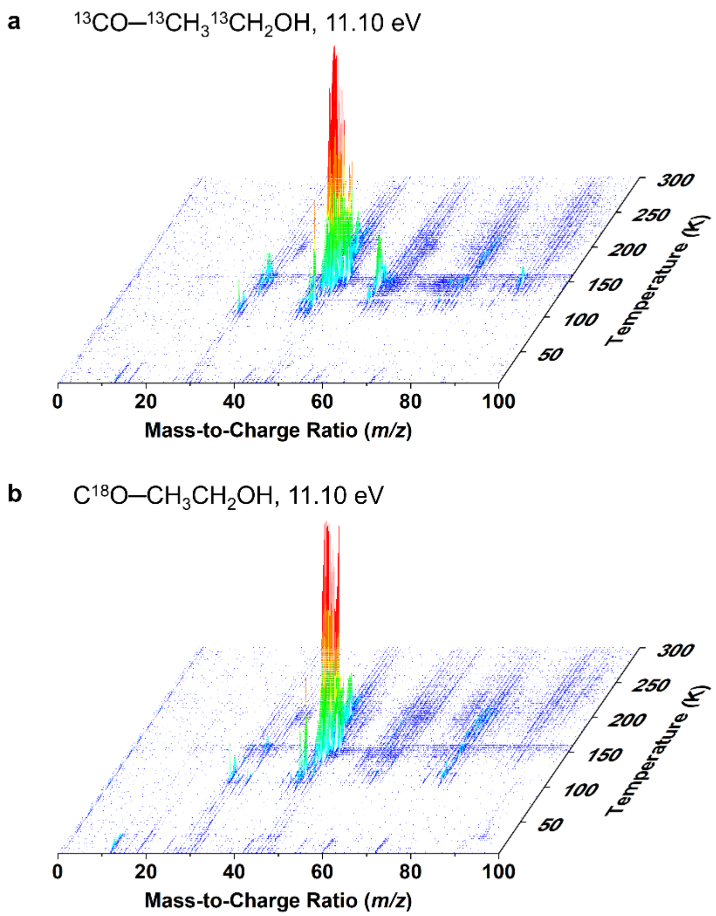

Supplementary Figure 7. PI-ReToF-MS data during TPD of carbon monoxide–ethanol ices with low dose irradiation. Data were recorded at 11.10 eV for the irradiated ^{13}CO–$^{13}CH_3{}^{13}CH_2OH$ ice (**a**) and $C^{18}O$–CH_3CH_2OH ice (**b**).

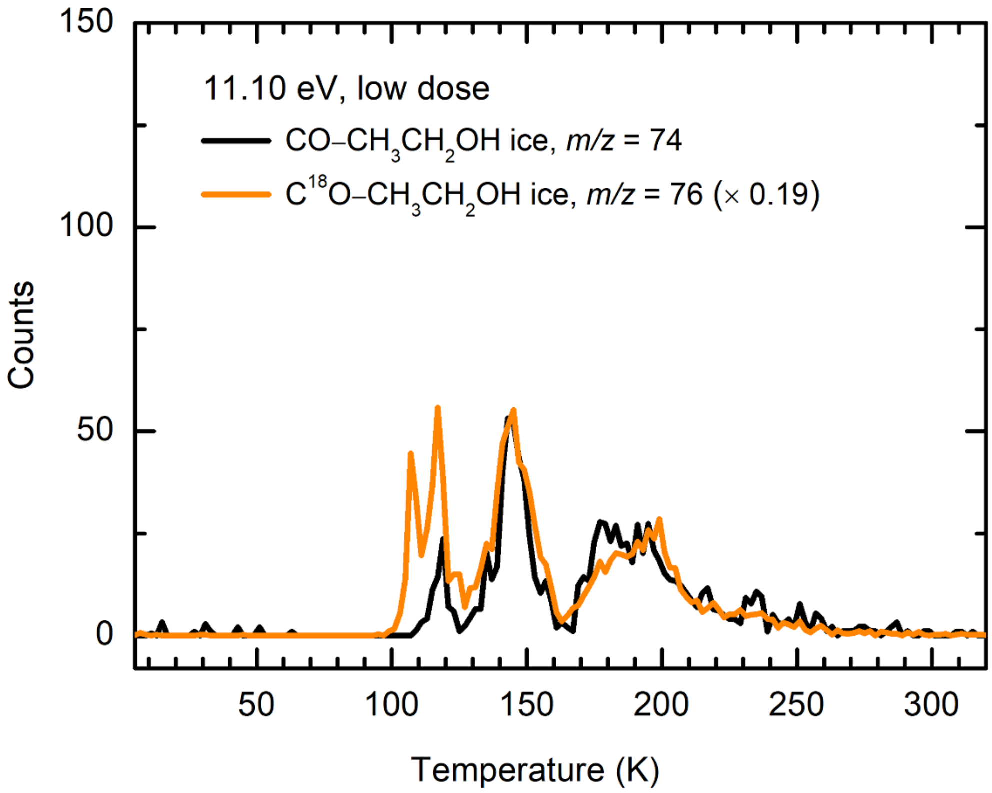

Supplementary Figure 8. TPD profiles of low dose irradiated carbon monoxide–ethanol ices. Data were recorded for the irradiated CO–CH_3CH_2OH ice and $C^{18}O$–CH_3CH_2OH ice at 11.10 eV. The first sublimation event peaking at 108 K in irradiated $C^{18}O$–CH_3CH_2OH ice is likely due to the likely due to the molecules that cosublimate with ketene (H_2CCO).

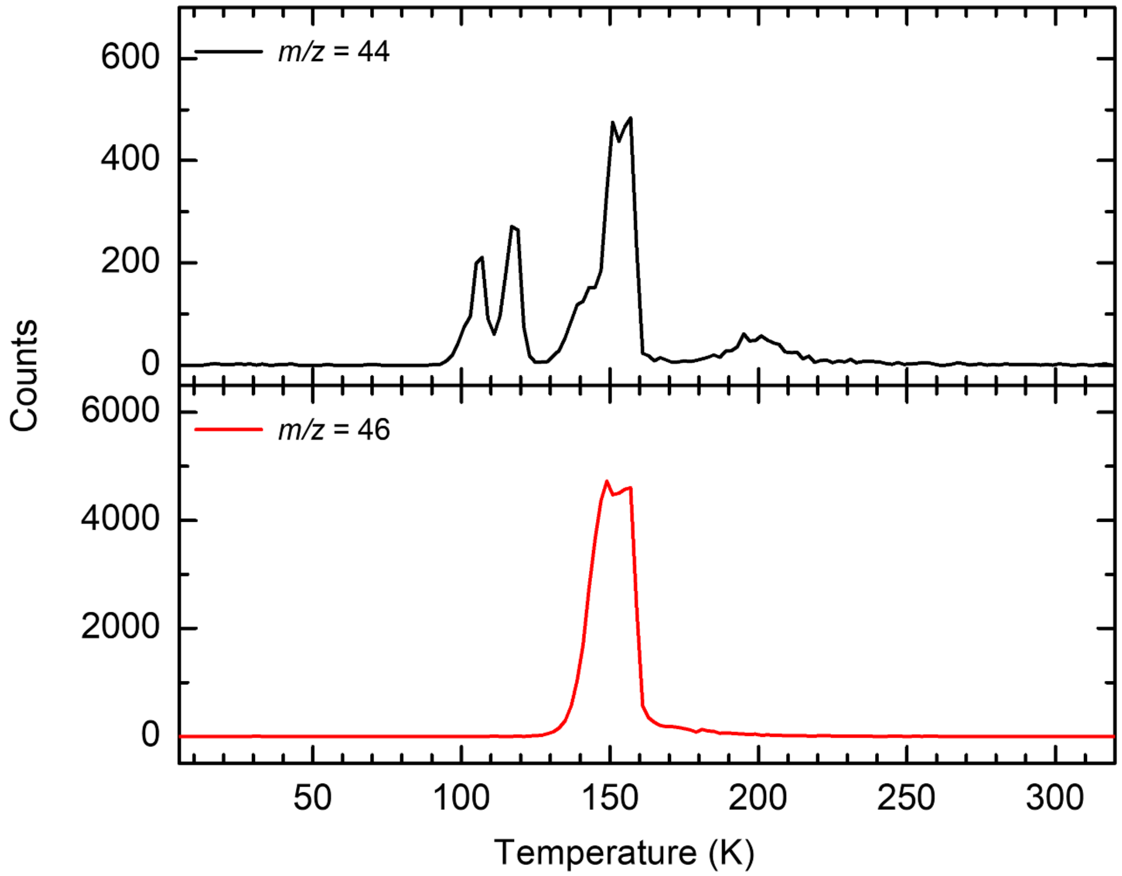

Supplementary Figure 9. TPD profiles of *m/z* = 44 (top) and *m/z* = 46 (bottom) in irradiated CO–CH_3CH_2OH ice. TPD profiles were measured at 11.10 eV with low dose (23 nA, 5 minutes) irradiation.

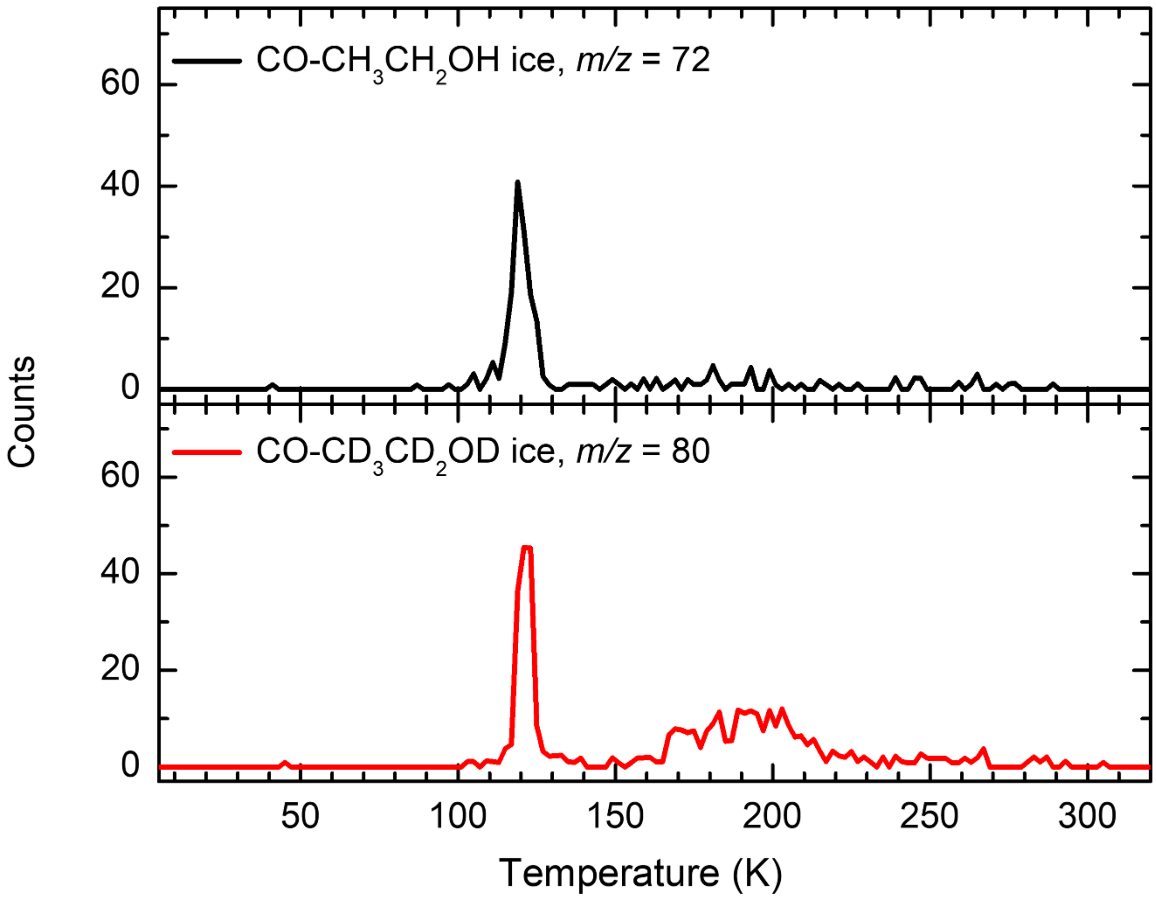

Supplementary Figure 10. TPD profiles of C_4H_8O isomers recorded at 9.29 eV in high dose experiments. TPD profiles of *m/z* = 72 in irradiated CO–CH_3CH_2OH ice (top) and of *m/z* = 80 in irradiated CO–CD_3CD_2OD ice were recorded at 9.29 eV with high dose (123 nA, 10 minutes) irradiation, confirming the formula C_4H_8O for the sublimation event peaking at 121 K.

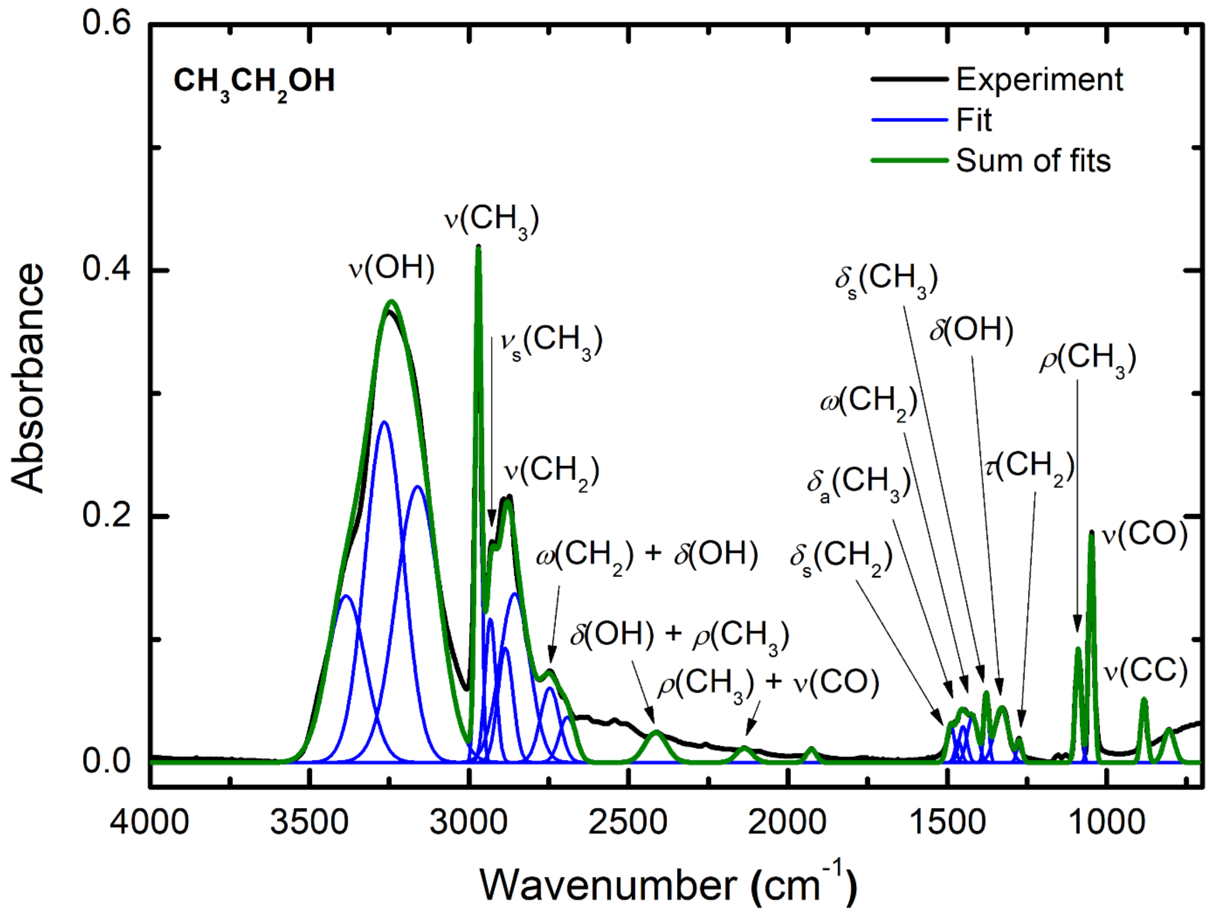

Supplementary Figure 11. Infrared spectrum of pure ethanol (CH_3CH_2OH) ice with a thickness of 760 ± 50 nm. The spectrum was measured immediately after deposition at 5 K. Detailed assignments are compiled in Supplementary Table 13.

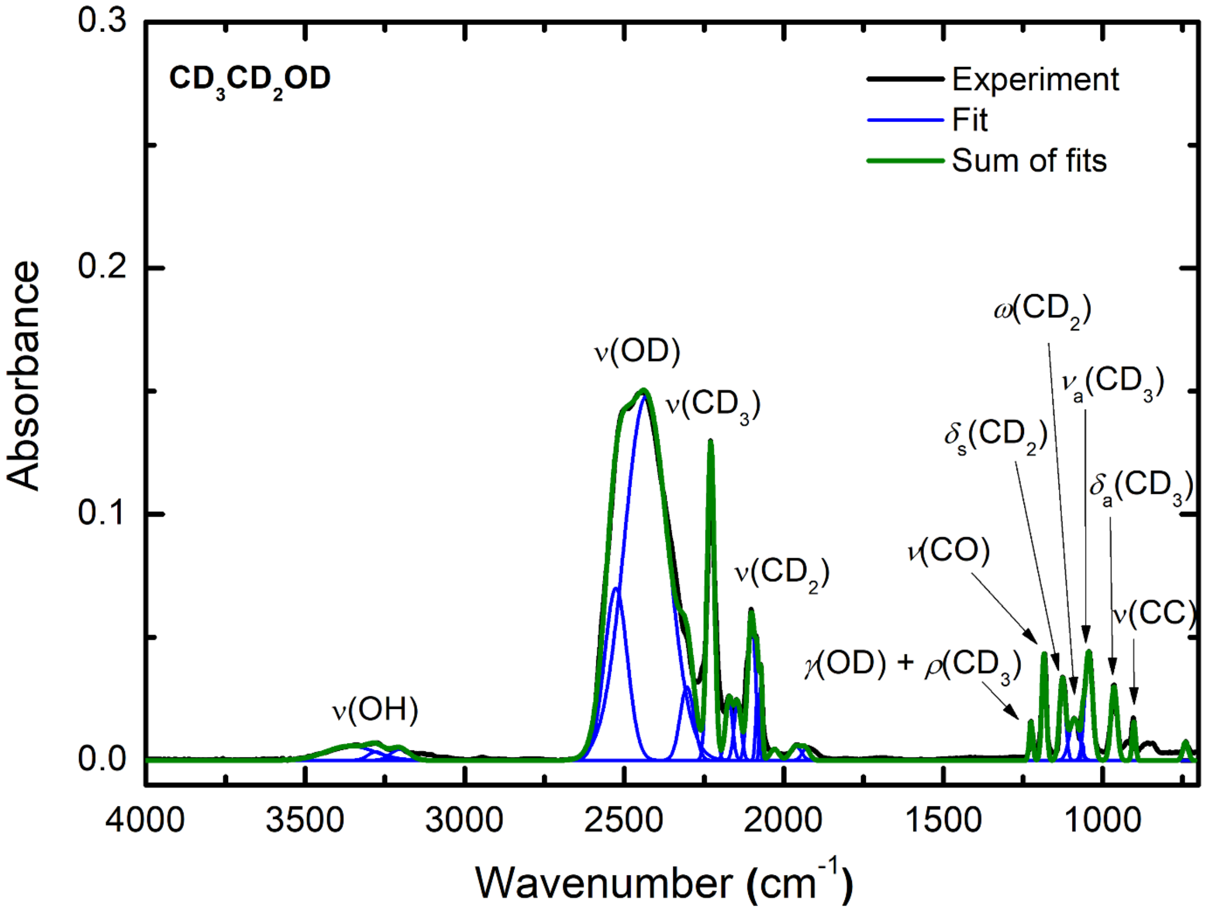

Supplementary Figure 12. Infrared spectrum of pure ethanol-d$_6$ (CD_3CD_2OD) ice with a thickness of 810 ± 50 nm. The spectrum was measured immediately after deposition at 5 K. Detailed assignments are compiled in Supplementary Table 14.

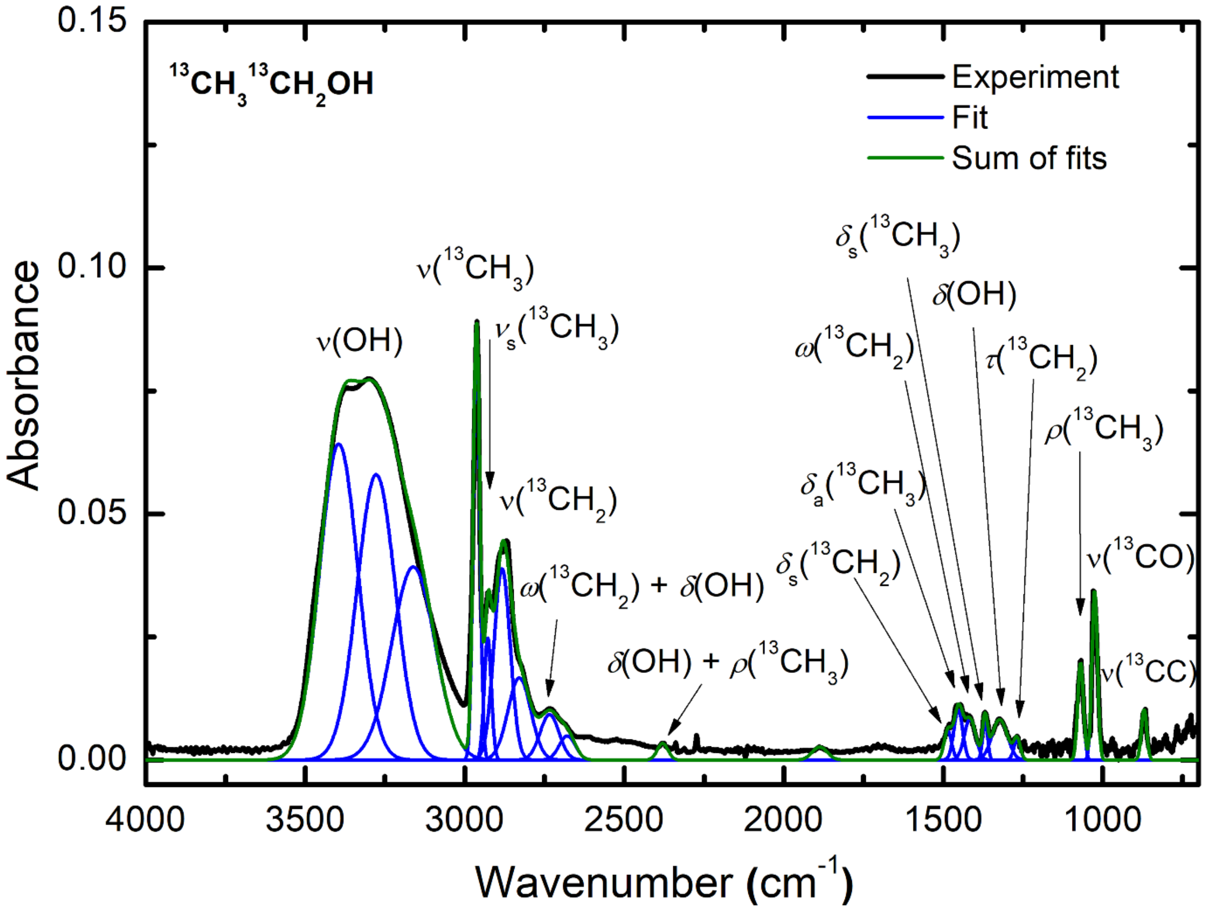

Supplementary Figure 13. **Infrared spectrum of pure ethanol-$^{13}C_2$ ($^{13}CH_3{}^{13}CH_2OH$) ice with a thickness of 450 ± 50 nm.** The spectrum was measured immediately after deposition at 5 K. Detailed assignments are compiled in Supplementary Table 15.

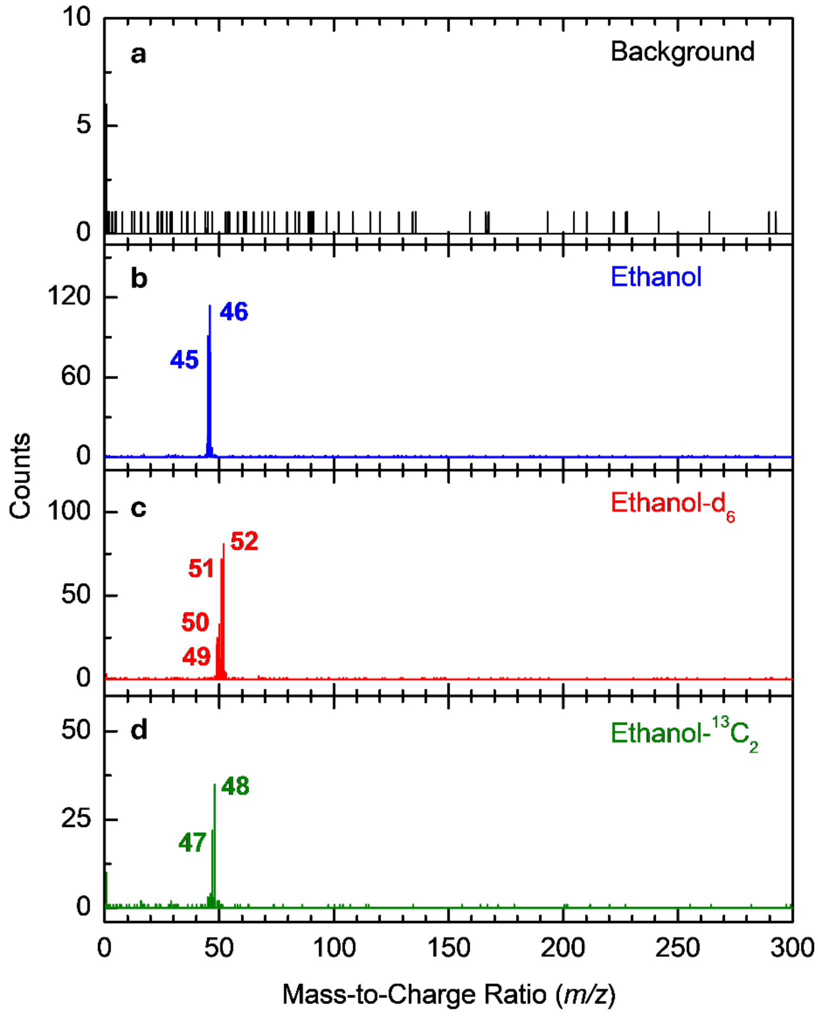

Supplementary Figure 14. Gas phase mass spectra of ethanol samples recorded at 11.10 eV. Mass spectra were collected for background gases with 9082 VUV laser pulses (**a**), ethanol at a pressure of 1 $\times 10^{-9}$ Torr with 2832 laser pulses (**b**), ethanol-d_6 at a pressure of 1 $\times 10^{-9}$ Torr with 9027 laser pulses (**c**), and ethanol-$^{13}C_2$ at a pressure of 4 $\times 10^{-10}$ Torr with 9020 laser pulses (**d**).

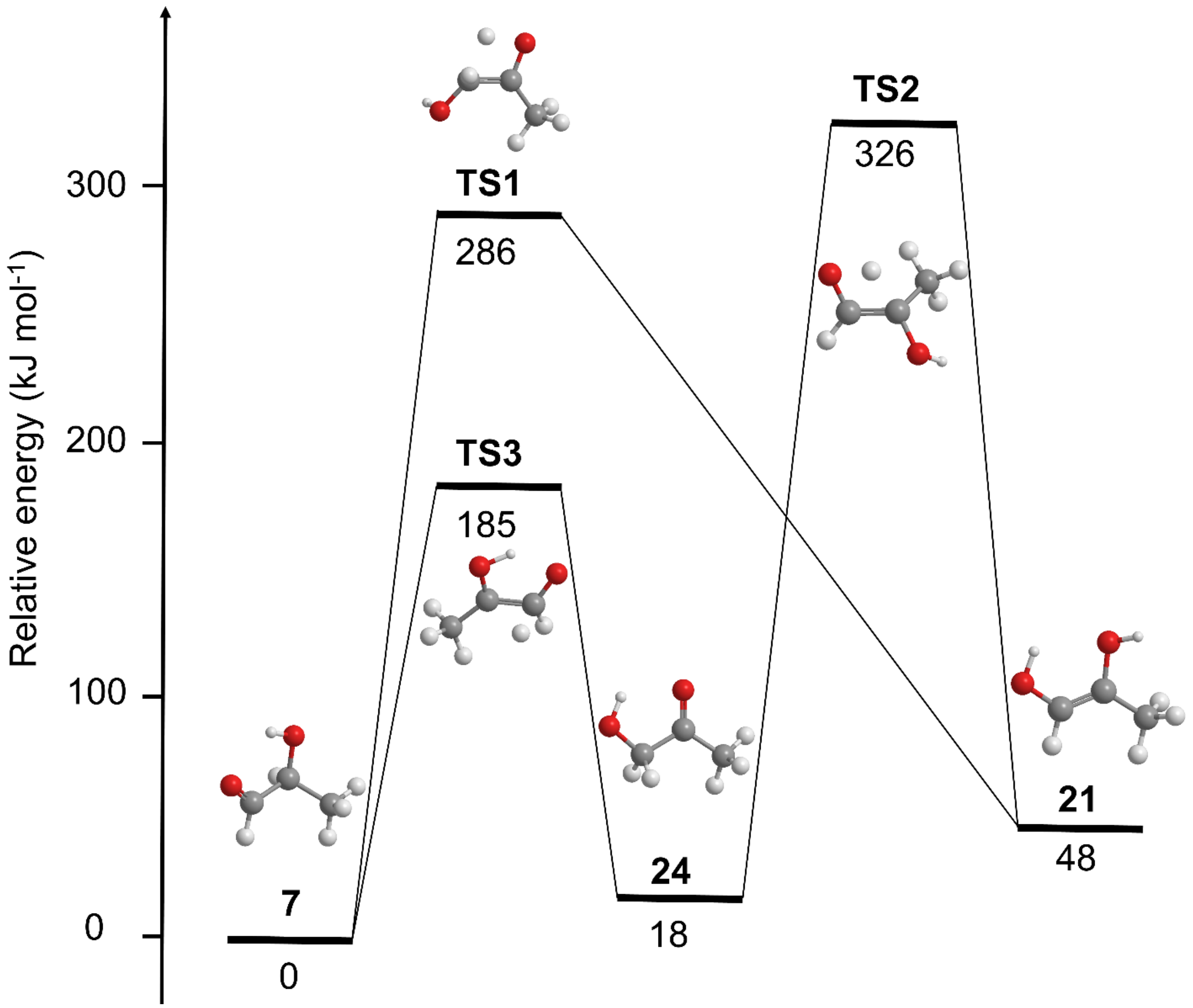

Supplementary Figure 15. Potential energy surfaces of the isomerization of lactaldehyde. Isomerization of lactaldehyde (**7**) can lead to the formation of 1,2-propenediol (**21**) and hydroxyacetone (**24**). Energies computed at the composite CBS-QB3 level of theory are shown in kJ mol^{-1} and are relative to the energy of lactaldehyde (**7**).

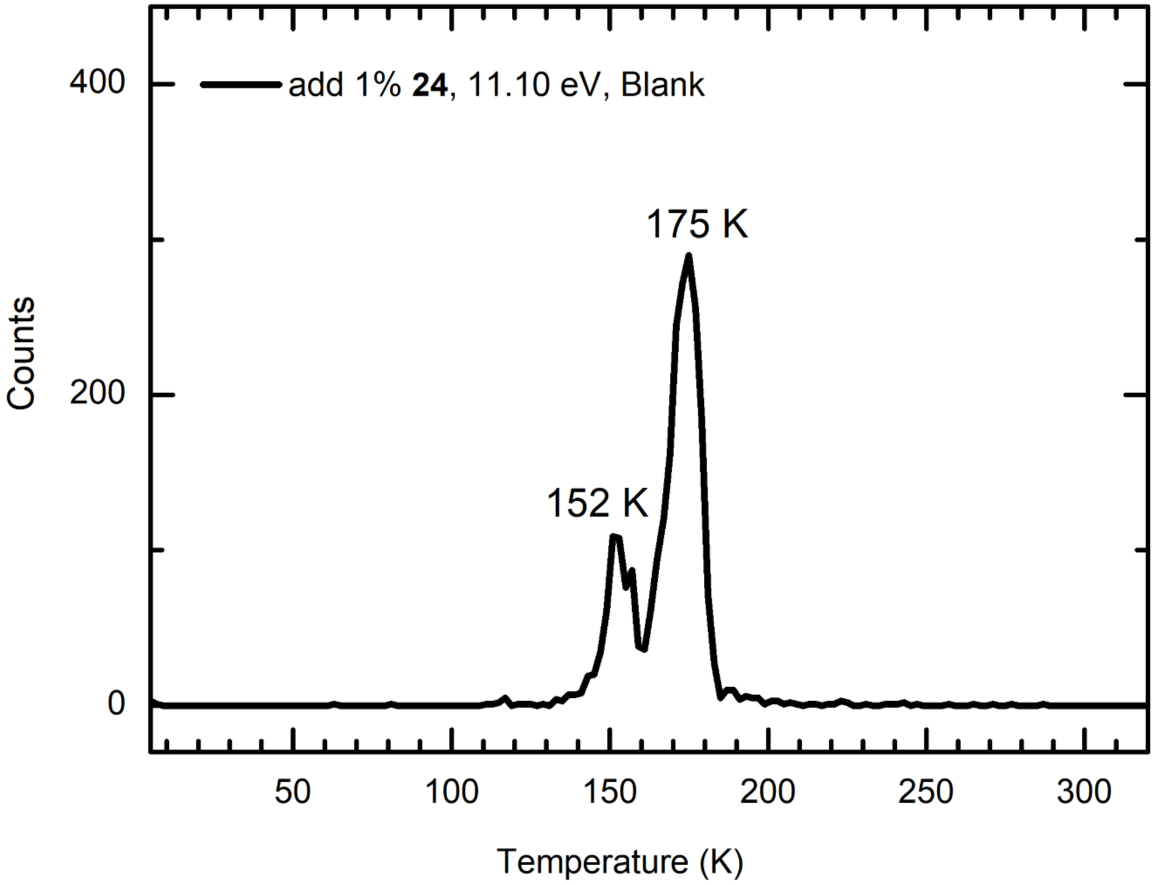

Supplementary Figure 16. Calibration experiment with hydroxyacetone. TPD profile of *m/z* = 74 in blank experiments with 1% hydroxyacetone (**24**) recorded at 11.10 eV under identical experimental conditions.

Supplementary Table 1. Absorption peaks observed in CO−CH_3CH_2OH ice before and after low dose irradiation (23 nA, 5 minutes) at 5 K. Vibration mode: stretching (v), bending (δ), wagging (ω), torsion (τ), rocking (ρ). Indication: asymmetric (a) and symmetric (s).

Pristine ice, absorptions before irradiation (cm^{-1})	
**CO**	Assignment[17]
4249	$2v(CO)$
2136	$v(CO)$
2090	$v(^{13}CO)$
**CH_3CH_2OH**	Assignment[18,19]
3608	$v(OH)_D$
3446, 3386, 3288	$v(OH)$
2977	$v_a(CH_3)$
2935	$v_s(CH_3)$
2900, 2876	$v_s(CH_2)$
2745	$\omega(CH_2) + \delta(OH)$
2415	$\delta(OH) + \rho(CH_3)$
1925	$v(CO) + v(CC)$
1488	$\delta_s(CH_2)$
1479	$\delta'_a(CH_3)$
1449	$\delta_a(CH_3)$
1418	$\omega(CH_2)$
1381	$\delta_s(CH_3)$
1325	$\delta(OH)$
1261	$\tau(CH_2)$
1090	$\rho(CH_3)$
1052, 1033	$v(CO)$
883	$v(CC)$
803	$\rho(CH_2)$
New absorptions after irradiation (cm^{-1})	Assignment[17,20-22]
2342	$v_3(CO_2)$
1853	$v_3(H\dot{C}O)$
1843	$v_2(HO\dot{C}O)$
1726	$v_2(H_2CO)$
1713	$v_4(CH_3CHO)$

Supplementary Table 2. Absorption peaks observed in CO−CH_3CH_2OH ice before and after high dose irradiation (123 nA, 10 minutes) at 5 K.

Pristine ice, absorptions before irradiation (cm^{-1})	
**CO**	Assignment[17]
4249	$2\nu(CO)$
2136	$\nu(CO)$
2090	$\nu(^{13}CO)$
**CH_3CH_2OH**	Assignment[18,19]
3609	$\nu(OH)_D$
3447, 3385, 3286	$\nu(OH)$
2977	$\nu_a(CH_3)$
2934	$\nu_s(CH_3)$
2900, 2875	$\nu_s(CH_2)$
2745	$\omega(CH_2) + \delta(OH)$
2415	$\delta(OH) + \rho(CH_3)$
1925	$\nu(CO) + \nu(CC)$
1489	$\delta_s(CH_2)$
1479	$\delta'_a(CH_3)$
1449	$\delta_a(CH_3)$
1418	$\omega(CH_2)$
1379	$\delta_s(CH_3)$
1324	$\delta(OH)$
1262	$\tau(CH_2)$
1090	$\rho(CH_3)$
1052, 1034	$\nu(CO)$
885	$\nu(CC)$
805	$\rho(CH_2)$
New absorptions after irradiation (cm^{-1})	Assignment[17,20-22]
2342	$\nu_3(CO_2)$
1853	$\nu_3(H\dot{C}O)$
1843	$\nu_2(HO\dot{C}O)$
1722	$\nu_2(H_2CO)$
1711	$\nu_4(CH_3CHO)$
1636	$\nu(C{=}O)/ \nu(C{=}C)$
1431	$\nu_7(CH_3\dot{C}HOH)$ (tentative)
1352	$\nu_9(CH_3\dot{C}HOH)$ (tentative)

Supplementary Table 3. Absorption peaks observed in CO−CD_3CD_2OD ice before and after low dose irradiation (23 nA, 5 minutes) at 5 K. Vibration mode: stretching (v), out-of-plane bending (γ), bending (δ), wagging (ω), rocking (ρ). Indication: asymmetric (a) and symmetric (s).

Pristine ice, absorptions before irradiation (cm^{-1})	
**CO**	Assignment[17]
4249	$2v(CO)$
2136	$v(CO)$
2090	$v(^{13}CO)$
**CD_3CD_2OD**	Assignment[23]
2664	$v(OD)_D$
2549, 2510, 2440	$v(OD)$
2233	$v_a(CD_3)$
2174	$v_s(CD_3)$
2105	$v_s(CD_2)$
2029	$\delta'_a(CD_3) + \delta_a(CD_3)$
1962	$\delta'_a(CD_3) + v(CC)$
1931	$v_a(CD_3) + v(CC)$
1227	$\gamma(OD) + \rho(CD_3)$
1184	$v(CO)$
1128	$\delta_s(CD_2)$
1090	$\omega(CD_2)$
1066	$\delta'_a(CD_3)$
1047	$v_a(CD_3)$
967	$\delta_a(CD_3)$
904	$v(CC)$
741	$\rho(CD_3)$
New absorptions after irradiation (cm^{-1})	Assignment[17,20,21,24]
2342	$v_3(CO_2)$
1796	$v_3(D\dot{C}O)$
1780	$v_2(DO\dot{C}O)$
1715	$v_4(CD_3CDO)$
1695	$v_2(D_2CO)$
1680	$v(C{=}O)$
1514	$v(C{=}C)$

Supplementary Table 4. Absorption peaks observed in CO−CD_3CD_2OD ice before and after high dose irradiation (123 nA, 10 minutes) at 5 K.

Pristine ice, absorptions before irradiation (cm^{-1})	
**CO**	Assignment[17]
4249	$2\nu(CO)$
2137	$\nu(CO)$
2090	$\nu(^{13}CO)$
**CD_3CD_2OD**	Assignment[23]
2664	$\nu(OD)_D$
2549, 2510, 2440	$\nu(OD)$
2233	$\nu_a(CD_3)$
2173	$\nu_s(CD_3)$
2105	$\nu_s(CD_2)$
2029	$\delta'_a(CD_3) + \delta_a(CD_3)$
1962	$\delta'_a(CD_3) + \nu(CC)$
1931	$\nu_a(CD_3) + \nu(CC)$
1228	$\gamma(OD) + \rho(CD_3)$
1184	$\nu(CO)$
1128	$\delta_s(CD_2)$
1089	$\omega(CD_2)$
1067	$\delta'_a(CD_3)$
1046	$\nu_a(CD_3)$
967	$\delta_a(CD_3)$
904	$\nu(CC)$
741	$\rho(CD_3)$
New absorptions after irradiation (cm^{-1})	Assignment[17,20,21,24]
2342	$\nu_3(CO_2)$
2278	$\nu_3(^{13}CO_2)$
1796	$\nu_3(D\dot{C}O)$
1782	$\nu_2(DO\dot{C}O)$
1713	$\nu_4(CD_3CDO)$
1695	$\nu_2(D_2CO)$
1681	$\nu(C{=}O)$
1580	$\nu(C{=}O)$
1515	$\nu(C{=}C)$
993	$\nu_5(D_2CO)$

Supplementary Table 5. Absorption peaks observed in ^{13}CO–$^{13}CH_3{}^{13}CH_2OH$ ice before and after low dose irradiation (22 nA, 5 minutes) at 5 K. Vibration mode: stretching (*ν*), bending (*δ*), wagging (*ω*), torsion (*τ*), rocking (*ρ*). Indication: asymmetric (*a*) and symmetric (*s*).

Pristine ice, absorptions before irradiation (cm^{-1})	
**^{13}CO**	Assignment[17]
4155	$2\nu(^{13}CO)$
2137	$\nu(CO)$
2089	$\nu(^{13}CO)$
**$^{13}CH_3{}^{13}CH_2OH$**	Assignment
3608	$\nu(OH)_D$
3395, 3278, 3161	$\nu(OH)$
2963	$\nu_a(^{13}CH_3)$
2928	$\nu_s(^{13}CH_3)$
2883, 2827	$\nu_s(^{13}CH_2)$
2734	$\omega(^{13}CH_2) + \delta(OH)$
2679	$\delta_s(^{13}CH_3) + \delta(OH)$
2380	$\delta(OH) + \rho(^{13}CH_3)$
1890	$\nu(^{13}CO) + \nu(^{13}CC)$
1480	$\delta_s(^{13}CH_2)$
1450	$\delta_a(^{13}CH_3)$
1422	$\omega(^{13}CH_2)$
1369	$\delta_s(^{13}CH_3)$
1324	$\delta(OH)$
1269	$\tau(^{13}CH_2)$
1071	$\rho(^{13}CH_3)$
1026	$\nu(^{13}CO)$
871	$\nu(^{13}CC)$
New absorptions after irradiation (cm^{-1})	Assignment[20,22,25]
2276	$\nu_3(^{13}CO_2)$
1814	$\nu_3(H^{13}\dot{C}O)$
1804	$\nu_2(HO^{13}\dot{C}O)$
1688	$\nu_2(H_2{}^{13}CO)$
1675	$\nu_4(^{13}CH_3{}^{13}CHO)$

Supplementary Table 6. Absorption peaks observed in $C^{18}O$−CH_3CH_2OH ice before and after low dose irradiation (24 nA, 5 minutes) at 5 K. Vibration mode: stretching (ν), bending (δ), wagging (ω), torsion (τ), rocking (ρ). Indication: asymmetric (*a*) and symmetric (*s*).

Pristine ice, absorptions before irradiation (cm^{-1})	
**$C^{18}O$**	Assignment[17,26]
4147	$2\nu(C^{18}O)$
2137	$\nu(CO)$
2085	$\nu(C^{18}O)$
**CH_3CH_2OH**	Assignment[18,19]
3602	$\nu(OH)_D$
3455, 3392, 3267	$\nu(OH)$
2976	$\nu_a(CH_3)$
2934	$\nu_s(CH_3)$
2900, 2873	$\nu_s(CH_2)$
2745	$\omega(CH_2) + \delta(OH)$
2414	$\delta(OH) + \rho(CH_3)$
1925	$\nu(CO) + \nu(CC)$
1486	$\delta_s(CH_2)$
1452	$\delta'_a(CH_3)$
1419	$\omega(CH_2)$
1381	$\delta_s(CH_3)$
1327	$\delta(OH)$
1259	$\tau(CH_2)$
1090	$\rho(CH_3)$
1051	$\nu(CO)$
884	$\nu(CC)$
805	$\rho(CH_2)$
New absorptions after irradiation (cm^{-1})	Assignment[26]
2342	$\nu_3(CO_2)$
1808	$\nu_3(H\dot{C}^{18}O)$
1798	$\nu_2(HO\dot{C}^{18}O)$
1714	$\nu_4(CH_3CHO)$
1695	$\nu_2(H_2C^{18}O)$

Supplementary Table 7. Error analysis of adiabatic ionization energies (IEs) and relative energies (ΔE) of lactaldehyde (**7**) conformers; IEs and ΔE were computed at the composite CBS-QB3 level including the zero-point vibrational energy corrections. The IE ranges are corrected for the thermal and Stark effect by −0.03 eV and the combined error limits of −0.05/+0.05 eV.

Conformer	Structure	ΔE (kJ mol^{-1})	Computed IE (eV)	Corrected IE ranges (eV)
**7a**	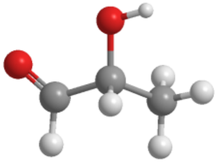	22.5	9.46	9.38 – 9.48
**7b**	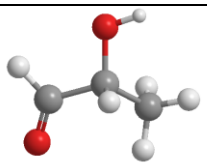	11.3	9.53	9.45 – 9.55
**7c**	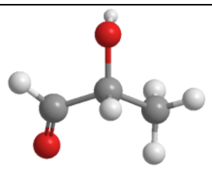	12.8	9.52	9.44 – 9.54
**7d**	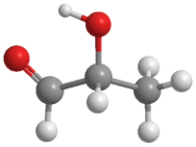	0.0	9.68	9.60 – 9.70
**7e**	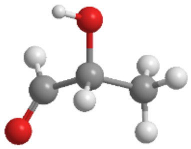	11.7	9.52	9.44 – 9.54

Supplementary Table 8. Error analysis of IEs and relative energies (ΔE) of 3-hydroxypropanal (**18**) conformers; IEs and ΔE were computed at the composite CBS-QB3 level including the zero-point vibrational energy corrections. The IE ranges are corrected for the thermal and Stark effect by −0.03 eV and the combined error limits of −0.05/+0.05 eV.

Conformer	Structure	ΔE (kJ mol^{-1})	Computed IE (eV)	Corrected IE ranges (eV)
**18a**	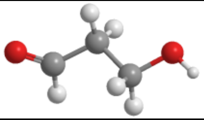	14.1	10.08	10.00 – 10.10
**18b**	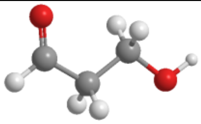	7.5	9.91	9.83 – 9.93
**18c**	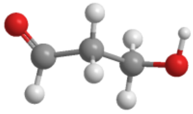	15.1	10.07	9.99 – 10.09
**18d**	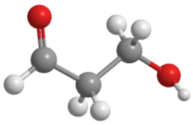	11.0	9.88	9.80 – 9.90
**18e**	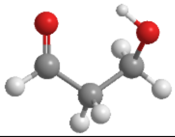	0.0	9.81	9.73 – 9.83

Supplementary Table 9. Error analysis of IEs and relative energies (ΔE) of ethyl formate (**19**) conformers; IEs and ΔE were computed at the composite CBS-QB3 level including the zero-point vibrational energy corrections. The IE ranges are corrected for the thermal and Stark effect by −0.03 eV and the combined error limits of −0.05/+0.05 eV.

Conformer	Structure	ΔE (kJ mol^{-1})	Computed IE (eV)	Corrected IE ranges (eV)
**19a**	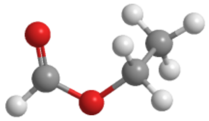	1.5	10.64	10.56 – 10.66
**19b**	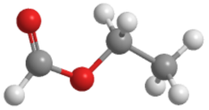	0.0	10.67	10.59 – 10.69
**19c**	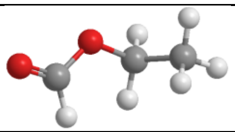	19.6	10.54	10.46 – 10.56

Supplementary Table 10. Error analysis of IEs and relative energies (ΔE) of *syn*-1,3-propenediol (**20**) conformers; IEs and ΔE were computed at the composite CBS-QB3 level including the zero-point vibrational energy corrections. The IE ranges are corrected for the thermal and Stark effect by −0.03 eV and the combined error limits of −0.05/+0.05 eV.

Conformer	Structure	ΔE (kJ mol^{-1})	Computed IE (eV)	Corrected IE ranges (eV)
**20a**	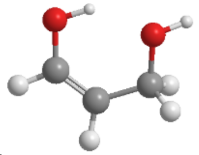	1.8	8.53	8.45 – 8.55
**20b**	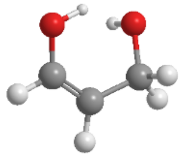	0.0	8.55	8.47 – 8.57
**20c**	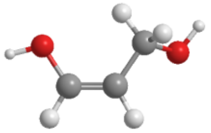	15.7	8.78	8.70 – 8.80
**20d**	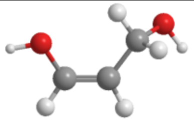	17.3	8.90	8.82 – 8.92
**20e**	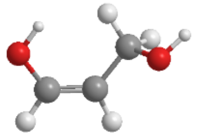	15.6	8.95	8.87 – 8.97
**20f**	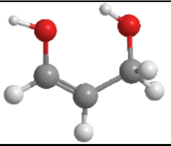	13.8	8.97	8.89 – 8.99
**20g**	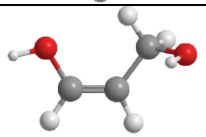	12.3	9.00	8.92 – 9.02
**20h**	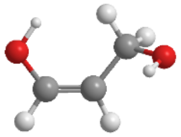	11.7	9.18	9.10 – 9.20

Supplementary Table 11. Error analysis of IEs and relative energies (ΔE) of *anti*-1,3-propenediol (**20**) conformers; IEs and ΔE were computed at the composite CBS-QB3 level including the zero-point vibrational energy corrections. The IE ranges are corrected for the thermal and Stark effect by −0.03 eV and the combined error limits of −0.05/+0.05 eV.

Conformer	Structure	ΔE (kJ mol^{-1})	Computed IE (eV)	Corrected IE ranges (eV)
**20i**	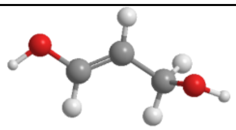	8.68	8.81	8.73 – 8.83
**20j**	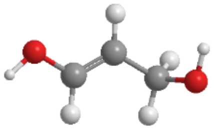	8.41	8.91	8.83 – 8.93
**20k**	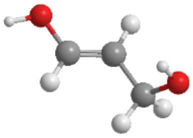	4.12	9.04	8.96 – 9.06
**20l**	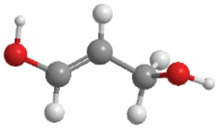	4.3	8.91	8.83 – 8.93
**20m**	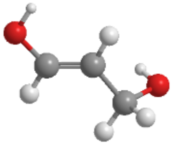	0.0	9.13	9.05 – 9.15

Supplementary Table 12. Error analysis of IEs and relative energies (ΔE) of 1,2-propenediol (**21**) conformers; IEs and ΔE were computed at the composite CBS-QB3 level including the zero-point vibrational energy corrections. The IE ranges are corrected for the thermal and Stark effect by −0.03 eV and the combined error limits of −0.05/+0.05 eV.

Conformer	Structure	ΔE (kJ mol^{-1})	Computed IE (eV)	Corrected IE ranges (eV)
**21a**	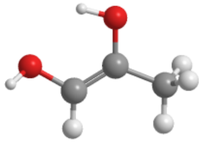	0.0	8.03	7.95 – 8.05
**21b**	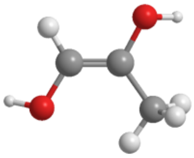	20.0	7.84	7.76 – 7.86
**21c**	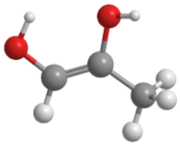	3.7	8.02	7.94 – 8.04
**21d**	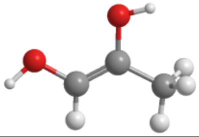	20.4	7.88	7.80 – 7.90
**21e**	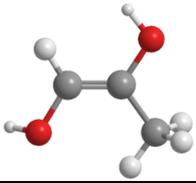	18.0	7.97	7.89 – 7.99
**21f**	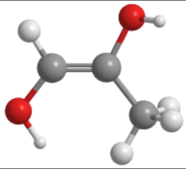	23.4	7.98	7.90 – 8.00
**21g**	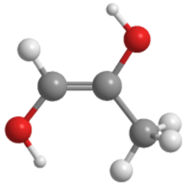	19.9	8.08	8.00 – 8.10

Supplementary Table 13. Absorption peaks observed in pure ethanol (CH_3CH_2OH) ice at 5 K.

Pristine ice, absorptions (cm^{-1})	
**CH_3CH_2OH**	Assignment[18,19]
3388, 3267, 3158	v(OH)
2971	$v_a(CH_3)$
2933	$v_s(CH_3)$
2887, 2857	$v_s(CH_2)$
2745	$\omega(CH_2) + \delta(OH)$
2690	$\delta_s(CH_3) + \delta(OH)$
2415	$\delta(OH) + \rho(CH_3)$
2136	$\rho(CH_3) + v(CO)$
1925	$v(CO) + v(CC)$
1490	$\delta_s(CH_2)$
1466	$\delta'_a(CH_3)$
1451	$\delta_a(CH_3)$
1421	$\omega(CH_2)$
1378	$\delta_s(CH_3)$
1328	$\delta(OH)$
1274	$\tau(CH_2)$
1089	$\rho(CH_3)$
1048	$v(CO)$
883	$v(CC)$
805	$\rho(CH_2)$

Supplementary Table 14. Absorption peaks observed in pure ethanol-d_6 (CD_3CD_2OD) ice at 5 K.

Pristine ice, absorptions before irradiation (cm^{-1})	
**CD_3CD_2OD**	Assignment[23]
2664	$\nu(OD)_D$
2527, 2430, 2304	$\nu(OD)$
2229	$\nu_a(CD_3)$
2172	$\nu_s(CD_3)$
2102, 2083	$\nu_s(CD_2)$
2029	$\delta'_a(CD_3) + \delta_a(CD_3)$
1962	$\delta'_a(CD_3) + \nu(CC)$
1931	$\nu_a(CD_3) + \nu(CC)$
1224	$\gamma(OD) + (CD_3)$
1183	$\nu(CO)$
1125	$\delta_s(CD_2)$
1088	$\omega(CD_2)$
1063	$\delta'_a(CD_3)$
1044	$\nu_a(CD_3)$
964	$\delta_a(CD_3)$
904	$\nu(CC)$
740	$\rho(CD_3)$

Supplementary Table 15. Absorption peaks observed in pure ethanol-$^{13}C_2$ ($^{13}CH_3{}^{13}CH_2OH$) ice at 5 K.

Pristine ice, absorptions (cm^{-1})	
**$^{13}CH_3{}^{13}CH_2OH$**	Assignment
3395, 3278, 3161	ν(OH)
2963	$\nu_a(^{13}CH_3)$
2928	$\nu_s(^{13}CH_3)$
2883, 2827	$\nu_s(^{13}CH_2)$
2734	$\omega(^{13}CH_2) + \delta(OH)$
2679	$\delta_s(^{13}CH_3) + \delta(OH)$
2380	$\delta(OH) + \rho(^{13}CH_3)$
1887	$\nu(^{13}CO) + \nu(^{13}CC)$
1486	$\delta_s(^{13}CH_2)$
1451	$\delta_a(^{13}CH_3)$
1416	$\omega(^{13}CH_2)$
1369	$\delta_s(^{13}CH_3)$
1323	δ(OH)
1268	$\tau(^{13}CH_2)$
1068	$\rho(^{13}CH_3)$
1025	$\nu(^{13}CO)$
870	$\nu(^{13}CC)$

Supplementary Table 16. Experimental conditions of carbon monoxide−ethanol ices including composition, ice thickness, irradiation parameters, and VUV photon energies.

Exp.	Ice	Composition of carbon monoxide to ethanol	Thickness (nm)	Current (nA)	Irradiation time (s)	Dose (eV/ carbon monoxide)	Dose (eV/ ethanol)	Photon energy (eV)
1	$CO-CH_3CH_2OH$	2.5 ± 0.4 : 1	880 ± 50	−	−	−	−	11.10
2	$CO-CH_3CH_2OH$	2.3 ± 0.3 : 1	880 ± 50	25 ± 3	300 ± 10	0.15 ± 0.04	0.34 ± 0.06	11.10
3	$CO-CD_3CD_2OD$	2.3 ± 0.7 : 1	880 ± 50	23 ± 1	300 ± 10	0.14 ± 0.03	0.35 ± 0.06	11.10
4	$CO-CH_3CH_2OH$	2.4 ± 0.3 : 1	880 ± 50	23 ± 1	300 ± 10	0.14 ± 0.03	0.31 ± 0.05	10.23
5[a]	$CO-CH_3CH_2OH$	2.5 ± 0.3 : 1	880 ± 50	23 ± 1	300 ± 10	0.14 ± 0.03	0.31 ± 0.05	10.23
6	$CO-CH_3CH_2OH$	2.3 ± 0.2 : 1	880 ± 50	24 ± 3	300 ± 10	0.15 ± 0.04	0.32 ± 0.06	9.71
7	$CO-CH_3CH_2OH$	2.3 ± 0.2 : 1	880 ± 50	22 ± 1	300 ± 10	0.13 ± 0.03	0.30 ± 0.05	9.29
8	$CO-CH_3CH_2OH$	2.2 ± 0.3 : 1	880 ± 50	126 ± 3	600 ± 10	1.53 ± 0.25	3.39 ± 0.55	9.29
9	$CO-CD_3CD_2OD$	2.4 ± 1.0 : 1	880 ± 50	123 ± 1	600 ± 10	1.50 ± 0.25	3.77 ± 0.61	9.29
10	$CO-CH_3CH_2OH$	2.1 ± 0.2 : 1	880 ± 50	121 ± 1	600 ± 10	1.47 ± 0.24	3.25 ± 0.53	8.25
11[b]	$CO-CH_3CH_2OH$	2.4 ± 0.4 : 1	880 ± 50	−	−	−	−	11.10
12[c]	$CO-CH_3CH_2OH$	2.4 ± 0.3 : 1	880 ± 50	−	−	−	−	11.10
13	$CO-CH_3CH_2OH$	2.4 ± 0.4 : 1	880 ± 50	123 ± 1	600 ± 10	1.50 ± 0.25	3.31 ± 0.54	11.10
14	$^{13}CO-^{13}CH_3{}^{13}CH_2OH$	1.4 ± 0.6 : 1	880 ± 50	22 ± 1	300 ± 10	0.14 ± 0.03	0.31 ± 0.05	11.10
15	$C^{18}O-CH_3CH_2OH$	1.3 ± 0.3 : 1	930 ± 50	24 ± 1	300 ± 10	0.16 ± 0.03	0.32 ± 0.05	11.10

[a] Repeat experiment 4.
[b] Add 1% ethyl formate (C_2H_5OCHO, **19**).
[c] Add 1% hydroxyacetone ($HOCH_2C(O)CH_3$, **24**).

Supplementary Table 17. Vacuum ultraviolet (VUV) light generation parameters. The uncertainty of VUV photon energies is less than 0.001 eV.

VUV photon energy (eV)	Nonlinear medium in four-wave mixing	ω_1 laser wavelength (nm)	ω_1 Dye	ω_2 laser wavelength (nm)	ω_2 Dye
11.10 ($2\omega_1 + \omega_2$)	Xenon	249.628	Coumarin 503	1064	–
10.23 ($2\omega_1 - \omega_2$)	Krypton	212.556	Stilbene 420	863.381	LDS 867
9.71 ($2\omega_1 - \omega_2$)	Krypton	212.556	Stilbene 420	633.856	DCM
9.29 ($2\omega_1 - \omega_2$)	Xenon	222.566	Coumarin 450	669.715	DCM in DMSO
8.25 ($2\omega_1 - \omega_2$)	Xenon	249.628	Coumarin 503	736.448	LDS 722

Supplementary Table 18. Comparison of the calculated IEs and relative energies (ΔE) of isomers **7**, **18**–**21** with their values from literature; IEs and ΔE were computed at the composite CBS-QB3 level including the zero-point vibrational energy correction. The relative energies (ΔE) are relative to the most stable conformer of ethyl formate (**19**).

Isomer	Our calculated ΔE (kJ mol^{-1})	Literature reported ΔE (kJ mol^{-1})[27]	Our calculated IE (eV)	Literature reported IE (eV)
Lactaldehyde (**7**)	39.8 – 62.3	35.6	9.46 – 9.68	
3-Hydroxypropanal (**18**)	51.6 – 66.8		9.81 – 10.08	9.85 – 9.95[28]
Ethyl formate (**19**)	0.0 – 19.6	0.0	10.54 – 10.67	10.60 – 10.62[29]
1,3-Propenediol (**20**)	88.7 – 106.0		8.53 – 9.18	8.57 – 8.89[24]
1,2-Propenediol (**21**)	65.6 – 89.0		7.84 – 8.08	7.81 – 7.85[24]

Supplementary Table 19. Mass-shifts of the potential species in carbon monoxide–ethanol ices upon isotopic substitution.

Formula	CO–CH_3CH_2OH	CO–CD_3CD_2OD (Fully deuterated)	^{13}CO–$^{13}CH_3{}^{13}CH_2OH$ (Fully carbon-^{13}C)	C^{18}O–CH_3CH_2OH
$C_3H_6O_2$	74	80	77	74, 76, 78
C_3H_6S	74	80	77	74
$C_2H_6N_2O$	74	80	76	74, 76
CH_6N_4	74	80	75	74
$C_4H_{10}O$	74	84	78	74, 76
$C_3H_{10}N_2$	74	84	77	74
$C_3H_{10}Si$	74	84	77	74
C_3H_3Cl	74	77	77	74
$C_2H_2O_3$	74	76	76	74, 76, 78, 80
C_6H_8	80	88	86	80
$C_4H_4N_2$	80	84	84	80
$C_3H_6F_2$	80	86	83	80
C_2H_5ClO	80	85	82	80, 82
C_2H_2ClF	80	82	82	80
CH_4O_2S	80	84	81	80, 82, 84
HBr	80	81	80	80

Supplementary References

1. Sun, J., So, S., da Silva, G. The gas phase aldose-ketone isomerization mechanism: Direct interconversion of the model hydroxycarbonyls 2-hydroxypropanal and hydroxyacetone. *Int. J. Quantum Chem.* **117**, e25434 (2017).
2. Arakawa, R. Mass spectral study of ionized-hydroxyacetone dissociation. *Bull. Chem. Soc. Jpn.* **64**, 1022-1024 (2006).
3. Simpson, J. A. Elemental and isotopic composition of the galactic cosmic rays. *Annu. Rev. Nucl. Part. Sci.* **33**, 323-382 (1983).
4. Kaiser, R. I., Maity, S., Jones, B. M. Synthesis of prebiotic glycerol in interstellar ices. *Angew. Chem. Int. Ed.* **54**, 195-200 (2015).
5. Umebayashi, T., Nakano, T. Fluxes of energetic particles and the ionization rate in very dense interstellar clouds. *Publ. Astron. Soc. Jpn.* **33**, 617 (1981).
6. Kaiser, R., Eich, G., Gabrysch, A., Roessler, K. Theoretical and laboratory studies on the interaction of cosmic-ray particles with interstellar ices. II. Formation of atomic and molecular hydrogen in frozen organic molecules. *Astrophys. J.* **484**, 487 (1997).
7. Wu, Q. T., et al. Role of low-energy (<20 eV) secondary electrons in the extraterrestrial synthesis of prebiotic molecules. *ACS Earth Space Chem.* **8**, 79-88 (2024).
8. Mason, N. J., Nair, B., Jheeta, S., Szymańska, E. Electron induced chemistry: a new frontier in astrochemistry. *Faraday Discuss.* **168**, 235-247 (2014).
9. Boyer, M. C., et al. The role of low-energy (≤ 20eV) electrons in astrochemistry. *Surf. Sci.* **652**, 26-32 (2016).
10. Turner, A. M., Kaiser, R. I. Exploiting photoionization reflectron time-of-flight mass spectrometry to explore molecular mass growth processes to complex organic molecules in interstellar and solar system ice analogs. *Acc. Chem. Res.* **53**, 2791-2805 (2020).
11. Arumainayagam, C. R., et al. Extraterrestrial prebiotic molecules: Photochemistry vs. radiation chemistry of interstellar ices. *Chem. Soc. Rev.* **48**, 2293-2314 (2019).
12. Kaiser, R., Roessler, K. Theoretical and laboratory studies on the interaction of cosmic-ray particles with interstellar ices. I. Synthesis of polycyclic aromatic hydrocarbons by a cosmic-ray-induced multicenter mechanism. *Astrophys. J.* **475**, 144 (1997).
13. Ferrari, B. C., Slavicinska, K., Bennett, C. J. Role of suprathermal chemistry on the evolution of carbon oxides and organics within interstellar and cometary ices. *Acc. Chem. Res.* **54**, 1067-1079 (2021).
14. Kleimeier, N. F., Eckhardt, A. K., Kaiser, R. I. Identification of glycolaldehyde enol (HOHC═CHOH) in interstellar analogue ices. *J. Am. Chem. Soc.* **143**, 14009-14018 (2021).
15. Maity, S., Kaiser, R. I., Jones, B. M. Infrared and reflectron time-of-flight mass spectroscopic study on the synthesis of glycolaldehyde in methanol (CH_3OH) and methanol–carbon monoxide (CH_3OH–CO) ices exposed to ionization radiation. *Faraday Discuss.* **168**, 485-516 (2014).
16. Kleimeier, N. F., Eckhardt, A. K., Schreiner, P. R., Kaiser, R. I. Interstellar formation of biorelevant pyruvic acid ($CH_3COCOOH$). *Chem* **6**, 3385-3395 (2020).
17. Bouilloud, M., et al. Bibliographic review and new measurements of the infrared band strengths of pure molecules at 25 K: H_2O, CO_2, CO, CH_4, NH_3, CH_3OH, HCOOH and H_2CO. *Mon. Not. R. Astron. Soc.* **451**, 2145-2160 (2015).
18. Burke, D. J., Wolff, A. J., Edridge, J. L., Brown, W. A. The adsorption and desorption of ethanol ices from a model grain surface. *J. Chem. Phys.* **128**, (2008).

19. Boudin, N., Schutte, W. A., Greenberg, J. M. Constraints on the abundances of various molecules in interstellar ice: laboratory studies and astrophysical implications. *Astron. Astrophys.* **331**, 749-759 (1998).
20. Eckhardt, A. K., et al. Formation of glyoxylic acid in interstellar ices: A key entry point for prebiotic chemistry. *Angew. Chem. Int. Ed.* **58**, 5663-5667 (2019).
21. Socrates, G. *Infrared and raman characteristic group frequencies: Tables and charts*, 3rd edn. John Wiley & Sons, Ltd. (2004).
22. Marks, J. H., et al. Complex reactive acids from methanol and carbon dioxide ice: Glycolic acid ($HOCH_2COOH$) and carbonic acid monomethyl ester (CH_3OCOOH). *Astrophys. J.* **942**, 43 (2023).
23. Mikawa, Y., Brasch, J. W., Jakobsen, R. J. Polarized infrared spectra of single crystals of ethyl alcohol. *Spectrochim. Acta A.* **27**, 529-539 (1971).
24. Wang, J., et al. Mechanistical study on the formation of hydroxyacetone (CH_3COCH_2OH), methyl acetate (CH_3COOCH_3), and 3-hydroxypropanal ($HCOCH_2CH_2OH$) along with their enol tautomers (prop-1-ene-1,2-diol ($CH_3C(OH)CHOH$), prop-2-ene-1,2-diol ($CH_2C(OH)CH_2OH$), 1-methoxyethen-1-ol ($CH_3OC(OH)CH_2$) and prop-1-ene-1,3-diol ($HOCH_2CHCHOH$)) in interstellar ice analogs. *Phys. Chem. Chem. Phys.* **25**, 936-953 (2023).
25. Wang, J., et al. Gas-phase detection of oxirene. *Sci. Adv.* **9**, eadg1134 (2023).
26. Maity, S., Kaiser, R. I., Jones, B. M. Formation of complex organic molecules in methanol and methanol–carbon monoxide ices exposed to ionizing radiation – A combined FTIR and reflectron time-of-flight mass spectrometry study. *Phys. Chem. Chem. Phys.* **17**, 3081-3114 (2015).
27. Alonso, E. R., et al. The laboratory millimeter and submillimeter rotational spectrum of lactaldehyde and an astronomical search in Sgr B2(N), orion-KL, and NGC 6334I. *Astrophys. J.* **883**, 18 (2019).
28. Rousso, A. C., Hansen, N., Jasper, A. W., Ju, Y. Identification of the Criegee intermediate reaction network in ethylene ozonolysis: impact on energy conversion strategies and atmospheric chemistry. *Phys. Chem. Chem. Phys.* **21**, 7341-7357 (2019).
29. P. J. Linstrom, Mallard, W. G. NIST Chemistry webBook, NIST Standard Reference Database Number 69, http://webbook.nist.gov/ (2013).
