# Supplementary material for: Interstellar formation of lactaldehyde, a key intermediate in the methylglyoxal pathway
Source: Nat Commun. 2024 Nov 24;15:10189. doi: 10.1038/s41467-024-54562-x (PMC11586434; doi:10.1038/s41467-024-54562-x)
Supplement: Supplementary file 2 — Description of Additional Supplementary Files [file 41467_2024_54562_MOESM2_ESM.pdf]

Description of Additional Supplementary Files

File name: Supplementary Data 1

Description: This file includes optimized cartesian coordinates, harmonic frequencies, and infrared intensities of $C_3H_6O_2$ isomers calculated at the composite CBS-QB3 level of theory.
