# Supplementary material for: Interstellar formation of lactaldehyde, a key intermediate in the methylglyoxal pathway
Source: Nat Commun. 2024 Nov 24;15:10189. doi: 10.1038/s41467-024-54562-x (PMC11586434; doi:10.1038/s41467-024-54562-x)
Supplement: Supplementary file 3 — Supplementary Data 1 [file 41467_2024_54562_MOESM3_ESM.docx]

**Supplementary Data 1.** Cartesian coordinates (Å), harmonic frequencies (cm^−1^), and infrared (IR) intensities (km mol^−1^) of C_3_H_6_O_2_ isomers calculated at the composite CBS-QB3 level of theory.

| **7a**  C -0.093004 0.043704 0.077398  H -0.284804 0.045761 1.152881  H 0.986776 -0.003239 -0.090217  H -0.533428 -0.861208 -0.350289  C -0.713004 1.292896 -0.564325  H -0.513589 1.266133 -1.651007  C -0.007508 2.54018 -0.056942  H 1.088309 2.533864 -0.263895  O -0.534063 3.454432 0.51265  O -2.093872 1.412743 -0.299153  H -2.526449 0.617577 -0.624204  Freq Int  106.2917 0.2032  241.8617 0.7295  267.0382 120.4627  280.1199 8.4017  352.1189 6.092  446.8479 6.9181  687.1678 27.6427  812.6845 61.2415  910.7748 7.7759  918.1345 3.3175  1056.0499 1.0323  1124.3744 6.3851  1171.9079 62.818  1235.2125 52.4018  1324.8832 12.1746  1401.3846 11.594  1413.1787 3.7488  1422.7664 7.1895  1494.5212 8.7533  1499.395 5.7832  1838.1944 176.5804  2841.9102 118.5383  2927.2151 58.0049  3027.1831 16.8151  3090.7616 26.5301  3106.6246 19.5382  3836.8912 29.1248 | **7a^+^**  C -0.138793 -0.005221 0.05443  H -0.514147 -0.016498 1.081127  H 0.95105 0.049565 0.066663  H -0.41357 -0.955134 -0.423359  C -0.751052 1.093485 -0.766215  H -0.348439 1.275998 -1.765339  C 0.040449 2.653338 -0.006224  H 1.072939 2.743211 -0.391844  O -0.56864 3.299239 0.732171  O -2.059009 1.279924 -0.765695  H -2.495422 0.924936 0.027181  Freq Int  52.6121 16.1715  187.1651 3.1645  220.4543 1.9708  233.0311 4.9982  321.2385 9.6552  429.9578 12.0166  512.5255 0.604  588.1951 127.2962  794.6994 12.8038  897.5362 27.415  990.663 33.0387  1044.7342 19.8313  1068.1619 28.3812  1162.266 14.0951  1241.3209 171.7934  1370.1926 13.2537  1400.2326 11.9682  1442.8443 75.0697  1466.8821 2.7316  1477.8361 20.9559  1981.8079 97.459  2949.4763 21.9789  3011.3457 23.3983  3084.1536 2.2456  3096.7934 9.5811  3127.8251 0.1697  3712.676 176.0514 |
| --- | --- |
| **7b**  C -0.072506 -0.019909 0.027411  H -0.255746 -0.08626 1.102288  H 1.005231 -0.034593 -0.14479  H -0.516889 -0.893969 -0.456601  C -0.670721 1.27009 -0.524282  H -0.532304 1.300139 -1.618826  C 0.039117 2.510193 0.005623  H -0.642676 3.367211 0.18884  O 1.225368 2.577616 0.192641  O -2.045271 1.445511 -0.194953  H -2.545436 0.722523 -0.583982  Freq Int  86.4897 12.0046  232.3118 2.8631  261.6108 40.9544  265.5445 85.4151  380.0477 7.6866  449.8879 10.7322  579.3011 5.661  824.3054 14.4122  914.9509 1.8572  948.4695 44.3194  1071.031 8.6301  1133.5343 67.5942  1139.4816 20.3963  1227.6645 54.5108  1338.2131 10.8991  1377.5458 16.6315  1401.886 15.6427  1414.8852 3.2406  1489.2188 8.3  1499.1398 4.6988  1822.2933 137.803  2902.5387 101.4014  2941.1448 41.4241  3033.5932 17.5157  3100.2662 23.1868  3119.7027 12.4172  3845.4197 32.7393 | **7b^+^**  C -0.096879 -0.077714 -0.054205  H -0.376582 -0.196331 0.996089  H 0.989264 -0.033032 -0.132265  H -0.443912 -0.959336 -0.606903  C -0.735152 1.121177 -0.680221  H -0.418475 1.395739 -1.692576  C 0.056981 2.655589 0.055745  H -0.604061 3.518397 -0.138597  O 1.083211 2.556701 0.579547  O -2.042968 1.334649 -0.540412  H -2.423262 0.842714 0.20717  Freq Int  72.097 19.6593  191.7715 4.3723  224.1879 1.3042  239.7598 7.7389  383.7575 6.9265  444.8008 7.3449  480.7721 12.9277  586.5332 131.2866  824.9567 3.92  905.2546 24.0713  992.7627 29.728  1030.7161 17.4705  1079.3663 33.638  1174.2282 18.3243  1229.8778 152.2792  1347.9192 7.3207  1403.0022 10.1174  1435.2708 81.2778  1465.6664 8.0424  1476.8355 13.6474  1966.6014 94.8623  2974.8782 26.2672  3023.3053 16.4932  3057.1025 13.5492  3093.3386 5.7942  3141.1886 0.6875  3710.2716 197.3501 |
| **7c**  C 0.100641 0.017259 -0.251456  H 0.407919 -0.136092 0.788174  H 1.006182 0.178075 -0.838614  H -0.404807 -0.883667 -0.602597  C -0.827726 1.218831 -0.363825  H -1.151527 1.341706 -1.405602  C -0.149718 2.534963 0.016937  H -0.872432 3.326486 0.314964  O 1.038348 2.726197 0.006811  O -2.040004 1.074759 0.372807  H -1.810078 0.845753 1.280127  Freq Int  80.0903 12.0707  226.7323 0.9389  258.4558 8.3982  343.1863 78.6619  399.0209 45.8955  440.044 12.5794  589.5889 5.0554  807.0838 14.4235  914.7738 2.467  957.9271 16.3559  1079.3628 64.0869  1121.844 9.2529  1138.1305 44.1878  1284.2295 8.6952  1332.8131 1.2476  1381.4347 1.7317  1405.6241 8.3187  1415.8424 46.9206  1489.8445 11.7102  1493.4684 2.9643  1815.3155 139.2093  2878.499 120.193  3014.4592 11.1152  3032.1206 20.6261  3099.7047 23.9065  3126.6983 11.8693  3808.8768 22.7245 | **7c^+^** (same as **7b^+^**)  C -0.096879 -0.077714 -0.054205  H -0.376582 -0.196331 0.996089  H 0.989264 -0.033032 -0.132265  H -0.443912 -0.959336 -0.606903  C -0.735152 1.121177 -0.680221  H -0.418475 1.395739 -1.692576  C 0.056981 2.655589 0.055745  H -0.604061 3.518397 -0.138597  O 1.083211 2.556701 0.579547  O -2.042968 1.334649 -0.540412  H -2.423262 0.842714 0.20717  Freq Int  72.097 19.6593  191.7715 4.3723  224.1879 1.3042  239.7598 7.7389  383.7575 6.9265  444.8008 7.3449  480.7721 12.9277  586.5332 131.2866  824.9567 3.92  905.2546 24.0713  992.7627 29.728  1030.7161 17.4705  1079.3663 33.638  1174.2282 18.3243  1229.8778 152.2792  1347.9192 7.3207  1403.0022 10.1174  1435.2708 81.2778  1465.6664 8.0424  1476.8355 13.6474  1966.6014 94.8623  2974.8782 26.2672  3023.3053 16.4932  3057.1025 13.5492  3093.3386 5.7942  3141.1886 0.6875  3710.2716 197.3501 |
| **7d**  C -0.057349 0.01931 0.085382  H -0.193878 0.036269 1.169136  H 1.011158 -0.04239 -0.140828  H -0.545554 -0.874041 -0.30646  C -0.694825 1.261387 -0.546493  H -0.509315 1.233969 -1.635082  C -0.028145 2.527428 -0.047422  H 1.071112 2.591163 -0.177055  O -0.65658 3.423782 0.46147  O -2.077402 1.298881 -0.291694  H -2.254685 2.172515 0.089843  Freq Int  122.835 0.076  232.5711 0.293  301.3124 18.4867  351.2251 11.8833  385.965 71.4739  480.9606 16.7087  705.3327 2.9512  827.4937 46.5217  911.0034 6.1705  928.9513 3.8537  1062.6984 50.1651  1127.0094 4.0702  1183.6028 41.2252  1274.853 27.5169  1338.4462 1.8325  1385.9014 50.5289  1405.1773 4.6698  1426.0194 48.1946  1487.2647 10.8323  1502.2098 3.0644  1797.5718 161.7663  2914.9532 58.5117  2937.1159 69.5646  3032.5218 18.2679  3102.0611 26.0446  3118.6626 14.599  3704.3183 69.2816 | **7d^+^**  C -0.05955 0.020208 0.090398  H -0.122849 -0.007716 1.179906  H 0.981087 0.039052 -0.234672  H -0.513417 -0.903838 -0.290888  C -0.850505 1.155264 -0.478485  H -0.750096 1.377675 -1.547289  C 0.186316 2.701245 0.050093  H 1.248773 2.498947 -0.178755  O -0.354333 3.609555 0.51247  O -2.054598 1.300261 0.044435  H -2.646291 1.857619 -0.486416  Freq Int  40.4648 18.758  184.1269 2.2983  205.119 2.6738  226.3125 7.1288  323.7746 5.3828  449.8822 28.2412  476.4888 37.1544  567.8421 88.8225  784.4202 12.1192  906.7766 12.7563  985.5199 1.6603  1041.4515 28.1324  1054.0903 24.5306  1137.8552 68.5489  1251.4509 5.6596  1313.9646 197.7875  1398.3005 14.2293  1447.3912 7.412  1469.1095 6.7074  1490.0716 22.047  1988.3439 115.1629  2952.7773 17.8575  3018.2931 26.7897  3046.6127 7.4746  3095.0147 5.1142  3142.3851 0.7699  3733.7156 223.0632 |
| **7e**  C -0.089217 0.008548 0.127071  H -0.191032 0.029981 1.215268  H 0.964627 -0.115967 -0.129793  H -0.653647 -0.84567 -0.251498  C -0.645683 1.290759 -0.483497  H -0.507077 1.279586 -1.571831  C 0.106026 2.495735 0.072171  H -0.278283 2.836891 1.060664  O 1.027627 3.038579 -0.478881  O -2.023807 1.365142 -0.125165  H -2.432196 2.080399 -0.623239  Freq Int  65.7963 11.2767  226.8302 0.0504  307.0588 14.2903  337.9232 97.7339  369.2911 18.5173  389.1825 22.6493  557.9493 5.5345  879.3774 13.6396  908.483 4.4672  979.6622 29.6427  1066.6802 43.2967  1115.0315 16.7487  1155.3636 14.7739  1279.2307 53.617  1308.8831 13.2662  1386.6424 10.3444  1391.287 3.3294  1412.231 7.8163  1482.9273 8.5149  1501.0686 2.5025  1817.6562 161.7157  2862.4736 106.6966  3015.3293 30.4507  3037.1622 13.0561  3107.422 21.2697  3119.3592 13.5833  3826.1954 31.0904 | **7e^+^**  C -0.120944 0.013722 0.159479  H -0.327999 0.007064 1.230762  H 0.952816 -0.034027 -0.027986  H -0.578713 -0.883432 -0.278224  C -0.756656 1.191518 -0.525277  H -0.560865 1.318645 -1.591777  C 0.236166 2.584681 0.130872  H 0.017661 2.716808 1.205315  O 0.944694 3.154079 -0.588101  O -2.005377 1.453204 -0.121738  H -2.523447 1.94172 -0.782056  Freq Int  60.4193 17.5319  207.2835 2.0302  213.2294 0.5581  271.6538 6.0991  348.5391 40.977  431.512 4.8298  533.6922 37.9575  584.0534 100.1769  840.4262 3.8333  898.2553 17.2126  994.503 5.3504  1052.3982 50.3538  1077.975 8.7751  1166.4336 60.8427  1238.8236 10.7542  1296.1331 156.4963  1396.7985 10.29  1442.2941 5.4941  1463.8615 9.4167  1476.9857 17.0371  1948.4384 87.4505  2971.3923 27.3805  3013.4733 32.2163  3095.3089 3.0011  3102.9895 5.5669  3143.2311 0.2091  3730.462 246.4542 |
| **18a**  C -1.058590 0.477415 0.141244  H -1.034099 1.329382 -0.554669  H -0.928372 0.870500 1.158849  C 0.078018 -0.488597 -0.186006  H -0.151035 -0.945198 -1.157717  H 0.147076 -1.279021 0.562580  C 1.412246 0.208470 -0.303389  H 1.443874 1.030458 -1.055985  O 2.392435 -0.067174 0.338966  O -2.275995 -0.248112 0.018779  H -2.999007 0.332442 0.273885  Freq Int  65.18 5.7803  99.96 7.8096  256.41 15.8345  277.16 109.6506  404.75 6.8350  526.16 32.5837  795.82 1.0579  867.36 1.5347  994.16 12.3974  1045.75 51.4891  1083.75 45.4864  1138.95 12.1493  1222.13 1.7151  1246.32 32.2721  1303.11 40.9927  1322.78 19.2457  1424.92 7.2659  1460.79 8.5132  1472.14 7.0979  1529.42 2.6656  1819.01 191.9553  2842.15 122.6861  2965.20 67.0522  2999.02 41.8784  3020.88 20.2321  3109.59 15.5560  3836.21 28.1819 | **18a^+^**  C 1.100283 0.524869 -0.196507  H 1.083107 1.399014 0.461932  H 1.027373 0.822113 -1.243687  C -0.064587 -0.440404 0.213842  H 0.069327 -0.764180 1.254566  H -0.131901 -1.315613 -0.432198  C -1.410553 0.321301 0.200403  H -1.446952 1.333407 0.650486  O -2.373506 -0.204496 -0.254418  O 2.250942 -0.206987 0.092464  H 2.628697 -0.617473 -0.701899  Freq Int  85.61 13.9216  119.46 15.8067  225.88 7.0772  371.52 7.3994  375.17 126.8965  458.42 11.5097  736.08 14.0452  834.92 57.3592  866.25 38.9483  932.55 32.3886  1042.24 107.7351  1104.76 11.5043  1113.78 10.0013  1227.20 99.0818  1235.94 8.1886  1261.64 2.2428  1353.38 13.1800  1369.10 36.3566  1446.13 16.1332  1501.74 52.2153  1783.92 20.4337  2927.84 13.3644  3031.32 11.8419  3038.78 3.1832  3116.42 7.7624  3143.03 2.8657  3737.16 607.3007 |
| **18b**  C -0.871163 -0.455231 0.000000  H -0.672062 -1.072948 0.884602  H -0.672062 -1.072948 -0.884602  C 0.053655 0.749165 0.000000  H -0.150071 1.384803 0.871792  H -0.150071 1.384803 -0.871792  C 1.519332 0.403050 0.000000  H 2.205478 1.278913 0.000000  O 1.959583 -0.719789 0.000000  O -2.205377 0.047170 0.000000  H -2.805803 -0.703578 0.000000  Freq Int  89.45 8.8068  182.23 3.9837  197.69 16.5585  286.52 128.5571  382.35 8.1075  693.27 1.2467  700.09 28.6770  893.08 0.0641  897.20 0.8161  1047.06 90.5941  1073.89 17.0140  1131.48 1.5807  1221.44 1.5944  1254.75 25.6545  1307.30 0.3776  1347.23 64.3227  1415.90 10.8079  1445.67 21.7914  1475.89 4.1900  1527.16 0.9160  1808.79 158.8607  2867.15 149.3954  2996.45 37.0208  3015.17 22.0067  3021.41 17.9018  3046.68 40.5579  3841.24 26.8757 | **18b^+^**  C -0.854932 -0.452243 0.186374  H -0.824433 -0.829383 1.209286  H -0.460102 -1.212141 -0.509510  C 0.028341 0.813940 0.011855  H -0.156903 1.586536 0.762906  H -0.119139 1.273136 -0.975845  C 1.497361 0.385738 0.032556  H 2.218735 1.227929 0.146452  O 1.889132 -0.729573 -0.089278  O -2.127996 -0.075122 -0.225596  H -2.771871 -0.093121 0.500998  Freq Int  72.04 6.1923  171.20 10.2965  194.69 8.7336  333.26 156.7321  379.19 2.0914  622.07 38.6041  709.19 16.7690  801.07 5.0954  859.03 35.5518  944.41 64.8418  1043.73 98.0132  1107.84 40.6226  1131.99 5.5277  1164.68 13.0250  1222.91 7.4660  1257.94 167.2987  1311.87 19.6508  1367.03 21.4828  1433.22 20.2959  1483.04 36.4847  1783.28 32.1539  2848.45 87.1041  2940.25 24.4269  3027.05 13.2612  3099.13 5.3780  3116.25 8.1807  3733.77 692.6824 |
| **18c**  C 1.045239 0.472367 0.185004  H 0.874898 0.751406 1.233185  H 1.037698 1.391599 -0.405907  C -0.073670 -0.470801 -0.291407  H -0.139962 -1.354558 0.348764  H 0.174715 -0.786527 -1.311678  C -1.421336 0.210410 -0.310441  H -1.503409 1.060388 -1.026035  O -2.355503 -0.095442 0.385242  O 2.335809 -0.089366 0.000412  H 2.412221 -0.855703 0.577497  Freq Int  63.64 10.8934  107.67 11.4919  258.39 7.4207  337.91 114.5738  401.89 16.7784  531.18 16.6228  802.36 2.8291  865.00 3.7779  965.72 7.0797  1057.86 137.5102  1077.03 53.2710  1108.84 3.2947  1191.75 1.1296  1264.30 27.6552  1317.80 7.4833  1392.92 3.2091  1419.42 38.2524  1429.37 20.2303  1470.60 7.0670  1517.00 1.1330  1815.47 186.0943  2851.83 125.4944  2996.11 44.0014  3024.40 16.0749  3069.36 7.1964  3092.15 40.4798  3826.17 26.3968 | **18c^+^** (Same as **18a^+^**)  C 1.100283 0.524869 -0.196507  H 1.083107 1.399014 0.461932  H 1.027373 0.822113 -1.243687  C -0.064587 -0.440404 0.213842  H 0.069327 -0.764180 1.254566  H -0.131901 -1.315613 -0.432198  C -1.410553 0.321301 0.200403  H -1.446952 1.333407 0.650486  O -2.373506 -0.204496 -0.254418  O 2.250942 -0.206987 0.092464  H 2.628697 -0.617473 -0.701899  Freq Int  85.61 13.9216  119.46 15.8067  225.88 7.0772  371.52 7.3994  375.17 126.8965  458.42 11.5097  736.08 14.0452  834.92 57.3592  866.25 38.9483  932.55 32.3886  1042.24 107.7351  1104.76 11.5043  1113.78 10.0013  1227.20 99.0818  1235.94 8.1886  1261.64 2.2428  1353.38 13.1800  1369.10 36.3566  1446.13 16.1332  1501.74 52.2153  1783.92 20.4337  2927.84 13.3644  3031.32 11.8419  3038.78 3.1832  3116.42 7.7624  3143.03 2.8657  3737.16 607.3007 |
| **18d**  C -0.875413 -0.466151 0.022854  H -0.697129 -1.050591 0.933364  H -0.658100 -1.119282 -0.822752  C 0.050453 0.748389 -0.012108  H -0.138749 1.417098 0.840544  H -0.159212 1.354414 -0.903412  C 1.521458 0.412251 -0.007566  H 2.203473 1.291504 -0.027426  O 1.966252 -0.707916 0.012712  O -2.244332 -0.095589 -0.104761  H -2.504636 0.367958 0.696986  Freq Int  87.76 6.3990  171.13 1.6856  193.99 5.3455  312.05 114.3408  389.10 16.2791  685.51 6.8652  689.93 20.9778  879.51 0.8998  897.85 17.4436  1054.07 39.6119  1063.80 81.3096  1097.57 4.0704  1186.30 12.4934  1296.57 32.4444  1323.54 5.5603  1395.37 4.3733  1414.66 16.5888  1424.14 55.5289  1454.11 5.6027  1512.36 1.4914  1809.92 157.6522  2862.61 151.6178  2996.79 19.7820  3013.67 28.2670  3033.48 23.8129  3105.78 22.4895  3828.34 21.3783 | **18d^+^** (same as **18b^+^**)  C -0.854932 -0.452243 0.186374  H -0.824433 -0.829383 1.209286  H -0.460102 -1.212141 -0.509510  C 0.028341 0.813940 0.011855  H -0.156903 1.586536 0.762906  H -0.119139 1.273136 -0.975845  C 1.497361 0.385738 0.032556  H 2.218735 1.227929 0.146452  O 1.889132 -0.729573 -0.089278  O -2.127996 -0.075122 -0.225596  H -2.771871 -0.093121 0.500998  Freq Int  72.04 6.1923  171.20 10.2965  194.69 8.7336  333.26 156.7321  379.19 2.0914  622.07 38.6041  709.19 16.7690  801.07 5.0954  859.03 35.5518  944.41 64.8418  1043.73 98.0132  1107.84 40.6226  1131.99 5.5277  1164.68 13.0250  1222.91 7.4660  1257.94 167.2987  1311.87 19.6508  1367.03 21.4828  1433.22 20.2959  1483.04 36.4847  1783.28 32.1539  2848.45 87.1041  2940.25 24.4269  3027.05 13.2612  3099.13 5.3780  3116.25 8.1807  3733.77 692.6824 |
| **18e**  C -4.5920168255 1.2703001095 -0.3584848258  C -3.8922687322 0.1011523719 0.2882733476  C -2.3903605464 0.3227613885 0.4808544843  O -4.0709069860 2.3388851525 -0.5786601523  O -2.1149601377 1.4207278059 1.3329766098  H -2.4315570559 2.2041594697 0.8667959220  H -5.6570849377 1.1042420772 -0.6279369397  H -4.0960804103 -0.7957018340 -0.3121366288  H -4.3805433945 -0.0685383379 1.2579652918  H -1.9130245285 0.4497357157 -0.5006250430  H -1.9473964452 -0.5574339189 0.9513779341    Freq Int  136.5208 9.4461  183.6747 1.3964  253.7633 7.3966  399.3001 9.9152  543.4387 136.448  632.0075 10.323  784.5955 1.5703  839.7848 12.9836  951.3048 8.1147  961.247 18.1018  1081.2463 99.2175  1107.3471 10.5221  1218.0624 10.8528  1248.6464 24.3937  1371.3063 20.4421  1394.5711 1.323  1416.8812 18.5162  1424.8915 22.557  1443.7272 57.3091  1508.1038 1.7561  1795.5652 145.3802  2885.5363 132.5634  2990.8949 48.0381  3007.1776 20.2478  3038.5563 21.6397  3083.4355 32.0063  3789.2963 59.5393 | **18e^+^**  C -4.6538473254 1.2232341262 -0.2780887996  C -3.9322992030 0.0055846801 0.2568509305  C -2.4617887375 0.3721226639 0.4470689323  O -4.2158632870 2.3394230932 -0.1721286256  O -2.4329480526 1.6243955373 1.1065359397  H -1.7188627994 2.1951622397 0.7776583755  H -5.6387990396 1.0871219216 -0.7647549525  H -4.0789089913 -0.8575427906 -0.3970263951  H -4.4331300671 -0.2207262294 1.2095937657  H -1.9159033198 0.3972603066 -0.4978700892  H -2.0038491773 -0.3757455486 1.1125609181  Freq Int  202.6966 4.0995  234.3335 12.4447  274.3021 10.3046  420.2623 64.1028  472.8412 10.5514  617.1363 12.7708  814.5721 17.2902  854.2663 3.968  900.6817 9.5788  918.5888 8.58  1013.97 44.4854  1065.2784 1.3656  1162.603 13.5058  1200.5472 6.0432  1237.6988 41.1113  1268.7022 45.8771  1312.7104 94.5794  1396.0927 23.11  1427.4714 28.1742  1465.0746 9.2483  1705.6573 14.2748  2945.993 47.9165  2981.8257 46.4033  3019.9754 15.1164  3101.1805 11.5277  3108.9576 4.362  3735.5914 355.8184 |
| **19a**  O -1.502437 -0.889121 -0.225642  C -1.331112 0.199295 0.250637  O -0.211912 0.934448 0.175479  C 0.904006 0.355842 -0.556018  C 1.747988 -0.533516 0.341324  H -2.082930 0.761310 0.823693  H 1.466303 1.219483 -0.910260  H 0.511181 -0.199194 -1.408666  H 2.091107 0.016592 1.220418  H 2.625743 -0.885806 -0.207772  H 1.178096 -1.404727 0.668235  Freq Int  74.5446 1.3478  227.0944 2.7851  260.9985 9.34  330.3827 12.6076  461.6106 15.473  753.7559 5.3993  819.2937 4.1504  849.3128 11.465  1007.4232 15.7505  1040.4002 0.1418  1117.5906 8.3046  1170.998 203.585  1209.252 166.5681  1333.63 16.2261  1395.792 0.2026  1404.9053 2.8272  1420.6257 11.2514  1488.5753 9.293  1493.6693 3.214  1508.9699 8.4214  1803.8641 281.0365  3025.5884 73.6402  3036.8971 14.4085  3072.4848 18.6637  3101.6646 27.3948  3110.3444 5.7091  3134.0864 27.9397 | **19a^+^**  O -1.193582 -0.995316 -0.170281  C -1.254170 0.226503 0.079676  O -0.292151 1.057104 0.147300  C 1.090095 0.622905 -0.230507  C 1.355421 -0.791646 0.183507  H -2.261996 0.630676 0.265398  H 1.713224 1.349296 0.294118  H 1.152467 0.788934 -1.305810  H 1.381138 -0.951341 1.261673  H 2.240662 -1.205379 -0.311152  H 0.512294 -1.453068 -0.216430  Freq Int  167.7707 8.403  216.2029 48.0113  283.069 4.4923  388.7588 35.8607  446.0126 25.519  707.6857 4.3883  733.0403 10.4899  780.5422 25.2901  919.6742 29.2879  991.5836 1.3315  1032.4028 33.0734  1129.9292 21.9195  1199.7847 5.5083  1225.8392 0.1741  1270.4337 9.7849  1300.5843 98.6984  1327.2454 103.9589  1391.6331 11.8698  1444.1402 11.0996  1477.4681 6.8029  1549.9611 100.7123  2497.0149 725.6493  3018.8749 85.1419  3049.7833 26.4139  3078.8187 22.806  3133.4779 13.1037  3148.3767 7.2433 |
| **19b**  O -1.932799 -0.630424 0.000024  C -1.410979 0.450126 0.000007  O -0.092112 0.688722 -0.000026  C 0.770011 -0.480238 -0.000042  C 2.202053 0.011925 0.000034  H -1.945458 1.411420 0.000018  H 0.539997 -1.081793 -0.882398  H 0.539928 -1.081873 0.882241  H 2.405597 0.617415 -0.885707  H 2.887182 -0.839767 0.000019  H 2.405531 0.617329 0.885849  Freq Int  53.3666 0.7167  225.5859 6.8899  236.5409 2.1489  347.9896 21.7087  380.0032 5.8848  788.8748 1.0287  812.853 0.7565  856.4259 15.1394  1025.7476 21.0859  1040.4363 0.0838  1132.7766 13.9462  1179.21 4.5595  1202.3013 407.9814  1298.1411 1.3436  1394.8098 2.3963  1399.0594 0.9284  1427.346 15.3607  1486.656 7.2726  1500.8795 2.8794  1521.1969 7.513  1806.7111 305.7171  3024.0248 74.0276  3038.437 15.3011  3052.4397 12.9182  3087.4859 4.1122  3106.0598 26.0498  3116.603 42.345 | **19b^+^**  O -1.899833 -0.675472 0.155407  C -1.368064 0.460760 0.043972  O -0.155854 0.709522 -0.153848  C 0.845257 -0.458425 -0.309833  C 2.153022 0.006903 0.246850  H -2.082979 1.300327 0.138864  H 0.852237 -0.641193 -1.383356  H 0.396004 -1.300593 0.228479  H 2.515909 0.891243 -0.284309  H 2.887033 -0.790542 0.086176  H 2.096010 0.212928 1.315739  Freq Int  40.5183 1.1476  157.3083 0.0325  234.9746 3.4617  306.8774 14.2945  404.5785 28.1608  572.6116 1.2843  681.7531 67.2109  813.7855 10.993  947.6782 26.1432  970.0189 1.6799  1114.5285 18.5524  1134.9323 26.4884  1182.384 7.939  1271.0718 1.7666  1316.2834 135.9061  1370.4455 7.839  1404.8942 75.0267  1438.8695 4.4337  1472.1166 10.3851  1477.0448 25.8808  1563.9883 125.8084  2957.2616 100.7958  3004.5511 180.2677  3029.7371 17.6496  3087.5296 0.2295  3126.5732 16.4154  3150.1728 0.6779 |
| **19c**  O -2.297121 0.311474 -0.084123  C -1.222381 -0.018673 0.314954  O -0.177797 -0.260669 -0.502130  C 1.086099 -0.609369 0.097743  C 2.052231 0.560409 0.024296  H -0.974293 -0.167571 1.383948  H 1.455764 -1.467224 -0.465890  H 0.925541 -0.927967 1.134028  H 2.171078 0.892870 -1.008764  H 3.032687 0.264147 0.407572  H 1.692876 1.405106 0.617163  Freq Int  48.8565 2.2146  160.8244 13.2439  228.5235 0.2398  376.9289 1.0739  406.0445 3.7878  625.966 6.948  807.7014 4.1665  920.4992 10.4975  1037.0419 10.2322  1045.7574 26.5375  1118.0486 126.0355  1119.61 126.3199  1221.9346 150.4009  1331.2791 51.2516  1395.8545 2.8463  1415.8687 6.5067  1429.0864 10.4353  1486.3468 6.9939  1496.9462 0.6187  1515.6666 4.9814  1851.1899 426.6269  2927.7459 72.7691  3023.6199 38.1015  3034.6771 12.3951  3087.5488 2.6763  3104.1398 34.1824  3116.275 33.5768 | **19c^+^**  O -2.369112 0.328654 0.013334  C -1.248456 -0.213409 0.211973  O -0.203376 0.104145 -0.396092  C 1.142751 -0.603363 -0.119135  C 2.156670 0.459347 0.183170  H -1.305362 -1.002347 0.989678  H 1.314335 -1.131028 -1.054862  H 0.955886 -1.300953 0.698697  H 2.251686 1.167184 -0.641246  H 3.123154 -0.042055 0.304755  H 1.934420 0.991354 1.108997  Freq Int  47.559 13.2379  175.371 13.7175  210.4785 0.3715  293.471 1.3606  372.1039 10.0434  471.3635 17.2746  724.2441 91.8424  817.8297 4.7827  949.3255 58.3132  981.8656 0.7978  1108.8723 8.3789  1149.773 5.704  1184.4315 11.7063  1276.3044 6.9112  1352.5383 130.6747  1379.441 22.1031  1412.5787 35.3838  1463.9271 12.1678  1487.0253 16.2413  1495.1828 11.6322  1596.0092 197.459  2932.0758 47.4841  3037.7356 16.7609  3081.7684 6.1372  3107.2082 7.6378  3134.7877 1.4733  3170.1391 0.5593 |
| **20a**  O -1.464667 -0.891412 0.070526  C -1.267495 0.443312 -0.032849  C -0.103093 1.102539 -0.039862  C 1.263096 0.500821 0.101227  O 1.208220 -0.916677 -0.152598  H -0.585735 -1.308310 0.064338  H -2.207540 0.977732 -0.121642  H -0.140206 2.181216 -0.138414  H 1.656975 0.679656 1.113666  H 1.956709 0.975717 -0.606236  H 2.016330 -1.321329 0.173770  Freq Int  76.3778 5.0117  249.799 105.0106  277.3287 6.2917  403.5272 5.1265  443.3859 9.4926  704.7686 146.1693  728.0404 0.6814  796.7714 6.7615  885.7489 2.4374  963.9267 5.6094  1015.3897 16.1385  1037.674 11.6952  1128.3148 159.5806  1223.1794 70.5728  1259.2921 75.8088  1275.8432 26.0563  1392.9291 5.3601  1456.1995 7.2421  1471.8818 31.1819  1515.8845 2.238  1727.6111 152.1286  2955.3103 78.4115  2989.1774 58.6243  3164.0234 7.3072  3185.308 22.7118  3646.3042 194.5752  3853.5346 34.3392 | **20a^+^**  O -1.384171 -0.876433 -0.000107  C -1.302068 0.404806 0.000054  C -0.084504 1.116138 0.000072  C 1.252531 0.496583 -0.000136  O 1.141152 -0.914836 0.000229  H -0.466725 -1.279494 -0.000160  H -2.258137 0.921509 0.000152  H -0.137630 2.199761 0.000153  H 1.806023 0.869271 0.880365  H 1.805492 0.868894 -0.881142  H 1.999382 -1.354949 -0.000285  Freq Int  125.297 44.3281  251.3656 127.9461  312.9492 7.1743  332.5958 8.5449  449.6905 9.3975  719.2303 11.8099  779.948 11.9173  813.1455 22.7583  874.2327 26.2373  950.3577 40.4271  1060.9797 13.5101  1068.4789 69.9894  1167.0229 39.2653  1220.6569 0.0973  1240.937 84.0101  1312.4393 144.5394  1396.3857 40.1458  1423.8047 38.8634  1458.2303 7.9502  1514.04 1.5767  1590.2052 156.9439  2936.6404 29.5781  2954.2672 1.5955  3148.1767 310.7896  3190.0791 79.6126  3199.6324 40.3087  3826.4436 201.431 |
| **20b**  O 1.430855 -0.882761 0.156415  C 1.259790 0.435491 -0.101710  C 0.098379 1.102622 -0.094883  C -1.219995 0.468001 0.256819  O -1.303426 -0.915704 -0.155374  H 0.544738 -1.280189 0.233512  H 2.200429 0.924064 -0.332113  H 0.114417 2.161675 -0.322776  H -2.053059 1.038590 -0.169093  H -1.376068 0.433452 1.340066  H -1.278933 -0.926553 -1.119284  Freq Int  128.4443 4.1863  249.348 3.7968  335.6871 28.0885  472.7404 111.9178  486.8362 40.7726  720.7107 49.3576  766.2775 18.2562  799.6479 32.9767  903.8025 20.1964  963.0441 7.5989  986.9237 66.6684  1011.3217 4.1899  1114.3473 124.1311  1198.4368 40.1035  1258.5993 81.5408  1362.6787 6.3226  1388.5283 18.3703  1419.662 23.4548  1453.2427 21.7748  1506.6957 1.0291  1706.5227 155.623  3014.4595 62.3792  3047.6792 29.2173  3168.3466 5.1217  3189.9764 22.2091  3618.0885 166.1428  3802.4037 27.2034 | **20b^+^** (same as **20a^+^**)  O -1.384171 -0.876433 -0.000107  C -1.302068 0.404806 0.000054  C -0.084504 1.116138 0.000072  C 1.252531 0.496583 -0.000136  O 1.141152 -0.914836 0.000229  H -0.466725 -1.279494 -0.000160  H -2.258137 0.921509 0.000152  H -0.137630 2.199761 0.000153  H 1.806023 0.869271 0.880365  H 1.805492 0.868894 -0.881142  H 1.999382 -1.354949 -0.000285  Freq Int  125.297 44.3281  251.3656 127.9461  312.9492 7.1743  332.5958 8.5449  449.6905 9.3975  719.2303 11.8099  779.948 11.9173  813.1455 22.7583  874.2327 26.2373  950.3577 40.4271  1060.9797 13.5101  1068.4789 69.9894  1167.0229 39.2653  1220.6569 0.0973  1240.937 84.0101  1312.4393 144.5394  1396.3857 40.1458  1423.8047 38.8634  1458.2303 7.9502  1514.04 1.5767  1590.2052 156.9439  2936.6404 29.5781  2954.2672 1.5955  3148.1767 310.7896  3190.0791 79.6126  3199.6324 40.3087  3826.4436 201.431 |
| **20c**  O 1.877918 -0.705446 -0.078180  C 1.320496 0.547861 -0.065947  C 0.025496 0.751973 0.160077  C -0.981667 -0.326466 0.396424  O -2.129735 -0.046810 -0.413910  H 2.783875 -0.644588 -0.390197  H 2.005456 1.374838 -0.231738  H -0.338270 1.772636 0.179366  H -1.265141 -0.341247 1.460807  H -0.545312 -1.302023 0.152321  H -2.812025 -0.681775 -0.177166  Freq Int  68.281 2.0957  214.2574 31.9537  240.5619 128.539  264.2942 94.9339  336.8218 5.0652  525.3668 11.4781  671.7362 19.819  757.7415 38.1085  941.567 11.5159  953.0156 6.5757  1021.8068 21.8282  1047.1426 142.9008  1117.4023 12.7338  1232.6152 12.2806  1241.1358 95.5075  1288.8114 102.776  1298.9449 87.7513  1407.5864 4.9122  1475.0158 3.4348  1515.8081 2.468  1755.0853 74.099  2950.1154 72.9879  3025.0814 33.0666  3144.0458 14.4375  3185.2797 13.1069  3832.5291 14.6504  3867.8081 88.7741 | **20c^+^**  O 1.903047 -0.643839 0.000069  C 1.384669 0.551877 0.000009  C -0.001991 0.702671 -0.000058  C -0.949951 -0.413614 -0.000140  O -2.252958 0.054780 0.000172  H 2.874949 -0.645129 0.000099  H 2.044773 1.415757 0.000014  H -0.398567 1.713702 -0.000103  H -0.720256 -1.068621 0.869829  H -0.720513 -1.068067 -0.870620  H -2.877466 -0.680770 -0.000006  Freq Int  66.7699 24.4271  194.1678 1.9122  298.7481 154.9858  339.181 17.2023  401.4744 0.0286  548.9807 36.0136  625.5924 113.3075  681.7094 63.8255  898.5844 1.1445  991.2614 2.2393  992.1383 37.6627  1129.5118 47.8785  1157.3858 114.4236  1190.1474 1.016  1202.5953 9.1313  1265.0831 189.824  1311.1469 272.1433  1349.9358 169.932  1378.9885 13.2127  1507.0016 26.025  1619.2013 214.5382  2844.0099 1.6531  2861.3783 102.7246  3169.3825 8.7343  3189.2072 13.9718  3727.053 524.9581  3813.6554 277.2675 |
| **20d**  O 1.836846 -0.719079 -0.052556  C 1.305883 0.541044 -0.112306  C 0.023615 0.790344 0.146439  C -1.003456 -0.248880 0.490233  O -2.080222 -0.307037 -0.453532  H 2.746398 -0.695241 -0.358690  H 2.000184 1.337527 -0.367494  H -0.299273 1.826258 0.101422  H -1.397113 -0.069410 1.501978  H -0.549609 -1.239049 0.483562  H -2.509825 0.553791 -0.458277  Freq Int  56.9327 1.9649  215.2453 28.7554  226.5678 107.091  286.7273 110.8994  320.0299 15.2035  553.2797 13.206  656.7698 14.8771  760.1762 46.2309  944.496 3.3189  954.3808 16.7241  1001.2989 48.5757  1039.9204 134.8559  1097.0916 39.6688  1196.4744 42.0285  1287.289 33.3899  1293.3243 181.2348  1364.6951 1.3464  1407.4835 38.9396  1456.3437 7.6299  1505.9345 1.347  1747.592 78.9324  2965.5658 77.8749  3114.2987 7.9342  3134.5568 7.6477  3160.8561 28.6493  3825.3515 19.8093  3871.2219 94.9916 | **20d^+^**  O 1.620655 -0.710389 0.107721  C 1.236072 0.488405 -0.215860  C -0.066431 0.895995 0.074359  C -1.090609 -0.010579 0.671215  O -1.587056 -0.544049 -0.570474  H 2.547356 -0.895240 -0.119545  H 1.947990 1.163720 -0.685456  H -0.370089 1.873628 -0.283091  H -1.851164 0.511397 1.249761  H -0.671111 -0.834395 1.242515  H -2.345975 -0.026535 -0.880451  Freq Int  86.2269 4.3374  221.1686 29.5684  263.6409 16.7256  425.88 61.683  436.754 103.3764  625.9571 117.6426  661.1636 25.6374  806.2501 14.7055  911.6139 21.4912  981.5358 10.4668  1000.5842 17.2093  1041.0882 31.6576  1140.8817 27.2358  1169.9614 37.0186  1274.165 61.1218  1339.9615 124.4148  1349.2671 106.0419  1361.6922 15.0677  1469.3457 19.8742  1508.522 10.9073  1609.6651 150.163  3102.4371 4.2509  3162.7361 4.8122  3172.269 0.0632  3201.5915 6.8345  3727.7659 510.0937  3753.9449 177.906 |
| **20e**  O 1.905547 -0.731643 0.060000  C 1.333277 0.498809 0.147596  C 0.066375 0.838733 -0.104531  C -1.022472 -0.088593 -0.537563  O -1.963978 -0.245014 0.529496  H 1.229589 -1.384922 -0.154590  H 2.067746 1.233500 0.456637  H -0.210182 1.875040 0.048203  H -0.613541 -1.067925 -0.838189  H -1.517820 0.326181 -1.426860  H -2.751425 -0.662307 0.165818  Freq Int  36.3099 2.0062  254.3867 4.1174  262.5196 36.2318  298.0653 83.1564  437.9732 94.6152  576.3497 22.0921  652.4352 5.0024  777.3204 29.0421  922.6392 28.4086  973.3636 2.1636  1026.8118 32.4348  1038.0665 127.9437  1128.4535 111.9897  1235.6733 23.4163  1243.2349 116.8667  1282.8134 25.6048  1349.8717 5.3567  1418.1461 12.4457  1466.3757 10.7633  1522.5472 1.218  1721.7296 153.6524  2930.5522 88.5123  2983.2668 61.6718  3174.1949 1.1699  3196.3262 17.3134  3803.3737 27.0897  3818.2215 9.0974 | **20e^+^**  O 2.089529 -0.549254 0.034213  C 1.399345 0.547913 0.000285  C 0.003475 0.671995 -0.027975  C -0.973343 -0.427558 -0.070879  O -2.253459 0.081360 0.085486  H 1.570836 -1.371375 0.060685  H 2.030804 1.431338 -0.008677  H -0.392044 1.684134 -0.037296  H -0.722968 -1.192266 0.695032  H -0.838588 -0.949676 -1.046367  H -2.913456 -0.613102 -0.029563  Freq Int  27.9596 28.0591  191.1091 6.9211  250.8349 1.2765  321.6051 116.0525  393.9361 6.5611  561.0766 9.9066  635.5601 107.1615  673.9323 31.418  911.3763 43.2651  970.4025 31.8014  1014.1781 16.8529  1128.9591 112.9148  1145.8886 186.0126  1198.3459 25.5312  1214.5491 101.564  1253.6285 0.5595  1335.021 237.6223  1395.5743 24.8048  1403.4601 11.8354  1498.1913 29.4075  1587.8156 265.018  2831.9943 40.6892  2868.072 42.4846  3176.661 19.2131  3197.231 12.4074  3710.4584 113.049  3813.5961 267.9894 |
| **20f**  O 1.375483 -0.828785 0.199799  C 1.220070 0.502346 -0.128823  C 0.028636 1.090806 -0.087782  C -1.256226 0.385605 0.281448  O -1.411079 -0.901098 -0.304714  H 2.263586 -1.118584 -0.022514  H 2.126488 1.030384 -0.407593  H -0.010511 2.147220 -0.327136  H -2.106709 0.977675 -0.063122  H -1.336483 0.324055 1.379417  H -0.606473 -1.394228 -0.108790  Freq Int  75.5437 6.5461  206.8101 46.1386  229.47 75.0488  369.3618 7.6963  449.7194 101.8806  514.7926 29.8757  718.2112 40.8501  767.3437 21.8965  883.9946 13.5287  919.8549 0.4134  1012.5054 16.5657  1029.9908 36.1819  1102.6858 114.4973  1226.6792 45.6603  1257.7991 14.5605  1282.6625 152.023  1367.99 1.2476  1420.9302 3.6328  1457.0877 69.7787  1504.0121 0.6018  1741.6593 51.117  2941.9536 78.1569  3074.2322 37.0563  3153.6192 6.9032  3183.2537 17.7306  3802.1829 53.1329  3868.7247 91.0024 | **20f^+^**  O 1.387935 -0.822990 0.036171  C 1.281515 0.481640 -0.034618  C 0.026683 1.076170 -0.002020  C -1.280293 0.415769 0.028097  O -1.423978 -0.949627 -0.086319  H 2.304017 -1.141165 -0.031541  H 2.186574 1.075871 -0.130059  H 0.007386 2.161378 0.025073  H -1.941066 0.891638 -0.717602  H -1.740212 0.786318 0.985780  H -0.695782 -1.434583 0.320776  Freq Int  120.1018 37.4686  139.6725 2.6031  208.9496 3.0093  331.2808 100.2501  428.4677 13.8744  594.7291 131.2045  701.2928 38.7513  794.8207 37.0379  853.0157 20.6358  863.9672 11.67  979.2847 0.6416  1094.214 42.9905  1170.9993 53.3773  1184.6978 186.8864  1243.8387 58.3211  1297.4069 130.981  1318.2624 56.7173  1335.4837 119.1449  1391.7338 0.748  1487.1591 25.6995  1602.8219 223.814  2735.2693 84.9867  2962.0587 34.9862  3170.6956 10.1311  3188.645 12.4952  3724.0598 430.2922  3803.9404 118.4096 |
| **20g**  O 1.843040 -0.716665 0.055214  C 1.301340 0.540745 0.104684  C 0.016668 0.780281 -0.156951  C -1.006793 -0.263789 -0.489093  O -2.155476 -0.181123 0.363222  H 2.760021 -0.677785 0.336793  H 1.988761 1.343745 0.357612  H -0.326286 1.808002 -0.108329  H -0.555223 -1.261361 -0.454386  H -1.397476 -0.111410 -1.499269  H -1.837596 -0.262310 1.268248  Freq Int  72.319 3.9017  228.229 2.9099  236.1059 96.9473  313.158 49.7945  376.4215 105.0346  539.888 3.8972  663.1606 11.6393  770.4677 42.316  933.0019 40.2149  951.6546 4.894  1017.3397 58.204  1035.617 131.2348  1121.4193 12.7543  1200.692 10.1909  1267.7391 5.3753  1290.9789 214.9333  1363.1402 1.5069  1409.0314 52.778  1452.6248 7.3701  1508.6081 1.3359  1743.52 74.8264  3021.9119 38.9106  3062.7822 29.9588  3139.4705 12.693  3176.7856 16.0709  3819.0032 15.9329  3870.3627 94.8261 | **20g^+^**  O 1.625172 -0.710687 -0.147442  C 1.241775 0.479200 0.226530  C -0.066551 0.892607 -0.014050  C -1.109963 0.043211 -0.663119  O -1.709058 -0.437199 0.553820  H 2.564370 -0.886639 0.029684  H 1.965054 1.143178 0.694848  H -0.372177 1.845485 0.402162  H -0.718327 -0.756201 -1.290103  H -1.855096 0.625298 -1.198731  H -1.304299 -1.278136 0.814943  Freq Int  74.7147 4.6796  220.8068 35.4321  259.0445 15.5121  415.645 32.5501  441.6073 115.4741  624.1253 119.255  659.2987 22.5396  807.8505 10.7474  919.0518 11.94  966.4594 47.8604  992.7037 20.0091  1042.1902 23.8715  1142.1683 28.8526  1171.2891 20.3218  1273.1085 61.4432  1335.7339 188.898  1345.4306 53.2636  1361.998 9.6809  1463.4086 17.9703  1506.6782 16.087  1609.1071 152.9889  3103.2816 3.188  3163.9428 5.2316  3173.342 0.2671  3209.0599 12.4063  3728.1728 427.2377  3757.9449 178.1367 |
| **20h**  O -1.962758 -0.662401 -0.122191  C -1.319795 0.536246 -0.108660  C -0.036041 0.780024 0.175744  C 1.011124 -0.240618 0.502377  O 2.129406 -0.181524 -0.384909  H -1.347953 -1.360946 0.130401  H -2.007745 1.333377 -0.365900  H 0.293782 1.812387 0.141399  H 0.589444 -1.260146 0.518068  H 1.429536 -0.065952 1.497334  H 1.778023 -0.161237 -1.281269  Freq Int  76.4798 2.9401  245.4615 16.4556  271.1688 16.461  361.1252 88.5502  449.6927 61.8921  562.5634 49.1906  653.6499 11.3851  782.0485 35.4013  924.4066 32.081  975.7334 6.0233  1016.0636 31.9135  1039.3332 167.061  1123.5173 104.5559  1208.6807 38.3954  1251.3544 44.6505  1334.7993 1.1813  1359.5766 15.5215  1413.6707 52.6697  1447.6298 18.658  1509.5693 1.2081  1714.5181 145.6288  2926.8834 92.1297  3057.9174 31.8072  3168.33 1.3927  3193.7304 15.098  3802.0611 24.844  3815.6696 19.313 | **20h^+^**  O -1.906715 -0.634685 -0.078037  C -1.305206 0.510617 -0.152878  C 0.028245 0.777925 0.188232  C 1.070784 -0.201876 0.616565  O 1.933591 -0.168771 -0.522080  H -1.350398 -1.366939 0.241251  H -1.965429 1.305128 -0.489287  H 0.370161 1.794891 0.029924  H 0.706915 -1.209379 0.843422  H 1.638385 0.158455 1.473488  H 1.622425 -0.774501 -1.209383  Freq Int  58.4032 16.6099  183.5651 20.3456  238.6347 26.162  351.875 98.2167  436.2185 58.7162  630.2647 19.6229  660.0684 47.7903  797.4569 52.6612  913.613 19.1854  990.4787 19.8211  1016.8882 43.6954  1028.7561 13.4421  1143.1495 30.1517  1171.2319 166.1756  1244.1579 64.1696  1331.6491 83.4901  1366.2815 8.674  1393.017 33.2487  1467.0782 7.8027  1501.3813 18.3059  1576.492 204.2517  3038.7427 12.7568  3128.1857 1.8035  3187.2919 9.4936  3202.3668 14.2103  3693.5602 97.9059  3775.2914 177.0118 |
| **20i**  C -0.076355 -0.077973 0.183022  H -0.518149 -0.472677 1.110048  H 1.000304 0.059401 0.363363  C -0.704448 1.232776 -0.153092  H -1.782608 1.261516 -0.273439  C 0.005383 2.344359 -0.325219  O -0.575102 3.548317 -0.608369  O -0.301931 -0.996132 -0.894703  H -0.099744 -1.879348 -0.571088  H 1.090161 2.344648 -0.235602  H 0.110334 4.20674 -0.744451  Freq Int  103.3543 3.9613  197.1706 27.6058  248.5775 80.4852  274.6362 118.4416  320.538 21.3004  448.1962 2.0242  554.3548 33.9639  835.6284 2.7934  939.5772 14.3503  954.0828 51.3256  1022.014 117.088  1112.5653 15.2141  1160.8393 30.0961  1211.759 131.5407  1239.6744 128.3381  1278.5966 39.288  1342.1206 52.5503  1375.3344 8.4075  1457.7441 19.1631  1512.3018 0.5145  1757.3635 113.1263  2957.5671 72.8202  2983.3468 66.3378  3126.6397 23.1635  3166.5318 9.1018  3823.8764 9.6878  3867.5881 93.389 | **20i^+^**  C -0.007421 -0.095311 0.006057  H 0.064298 -0.04657 1.116078  H 1.05532 -0.159957 -0.319958  C -0.563702 1.173902 -0.478798  H -1.45841 1.151155 -1.096751  C 0.005934 2.407766 -0.181246  O -0.558207 3.479975 -0.655705  O -0.774325 -1.157302 -0.439398  H -0.412617 -1.994455 -0.124262  H 0.901043 2.509938 0.430267  H -0.104066 4.302486 -0.405813  Freq Int  66.3838 1.1807  202.1082 1.6555  202.8327 7.7921  332.1276 135.5995  459.2439 3.8436  521.2196 6.7466  599.0762 97.7018  625.0654 74.1928  942.4153 7.6495  975.0227 0.0051  1045.8582 159.1681  1133.0499 272.015  1172.2656 27.9557  1191.4323 1.416  1217.4767 21.4952  1310.6068 81.6011  1315.0155 179.5778  1355.4344 177.9369  1361.3151 81.9339  1452.4345 23.759  1637.8869 175.3275  2837.53 0.3814  2852.7339 106.8871  3150.6794 2.1538  3171.2085 21.859  3723.5224 539.0414  3814.2479 271.8098 |
| **20j**  C -0.123728 -0.071235 0.253925  H -0.516491 -0.475107 1.198159  H 0.9591 0.021013 0.365745  C -0.735268 1.268472 -0.023958  H -1.817435 1.363781 0.023345  C -0.012592 2.337859 -0.348493  O -0.576864 3.552809 -0.612677  O -0.313303 -1.023211 -0.80189  H -1.259666 -1.092756 -0.96223  H 1.07282 2.28904 -0.412134  H 0.113695 4.184359 -0.828344  Freq Int  90.1431 5.9717  193.8009 36.0765  233.4227 115.8095  285.4857 73.6377  323.4645 35.0315  446.6848 7.1477  568.1158 40.567  837.7782 5.9399  940.6922 22.8257  953.1735 50.6793  1002.0312 161.7418  1080.2674 1.1917  1164.7181 26.316  1186.7336 45.7117  1247.0341 204.8938  1342.8365 41.0725  1366.6362 3.5143  1377.2994 9.5193  1423.1242 59.033  1504.9272 2.3245  1750.5961 115.8141  2975.3786 79.2937  3073.476 24.8498  3128.9909 18.8971  3141.2185 16.7203  3823.7231 20.8231  3865.0835 92.9801 | **20j^+^**  C -0.128273 -0.078787 0.392998  H -0.678887 -0.692246 1.104394  H 0.92761 -0.05907 0.6551  C -0.731697 1.269488 0.179331  H -1.796784 1.426975 0.323953  C 0.008751 2.295391 -0.405025  O -0.574808 3.429857 -0.647193  O -0.22392 -0.557663 -0.961602  H -1.094153 -0.955909 -1.11686  H 1.062355 2.175654 -0.651293  H 0.020073 4.101337 -1.022353  Freq Int  85.3808 15.1461  159.7704 8.6149  289.9328 30.0927  380.6736 48.8011  435.7244 70.6598  552.7906 62.9781  627.2412 150.5093  857.8559 26.8268  926.9668 9.3929  977.8038 4.4464  979.421 12.6219  1065.1101 54.8813  1161.599 49.7057  1181.0612 67.8829  1318.8665 19.1652  1341.7255 173.51  1356.4178 22.746  1367.657 116.4592  1388.1246 0.9239  1513.7481 7.9781  1622.0017 158.8505  3098.1011 5.9645  3157.0279 4.2422  3159.7195 1.3881  3180.6455 10.7134  3720.9387 490.6379  3752.6318 182.3483 |
| **20k**  C -0.123037 -0.115618 0.145284  H -0.508412 -0.53311 1.079458  H 0.966306 -0.009623 0.260007  C -0.739648 1.218312 -0.137937  H -1.822667 1.277467 -0.19055  C -0.013173 2.314099 -0.351924  O -0.576858 3.529158 -0.616912  O -0.4421 -1.094348 -0.849376  H -0.236955 -0.699901 -1.703506  H 1.07471 2.288828 -0.313662  H 0.116346 4.180362 -0.749693  Freq Int  111.9681 0.497  201.6065 32.8459  270.0038 50.6966  311.4627 43.8396  369.8682 115.2555  453.4405 11.9702  555.8239 23.674  835.1826 2.0435  932.1217 23.3593  958.5615 60.6758  1020.9017 152.6062  1109.6106 5.4324  1160.1824 49.0933  1189.7864 73.9076  1235.2122 111.1985  1334.8555 69.9414  1346.8275 10.7036  1387.3823 3.1993  1428.8939 75.3775  1507.1273 1.4302  1747.2513 105.2276  2967.6994 68.6291  3060.0326 36.7802  3122.7752 22.9557  3160.6789 8.7605  3814.934 15.0679  3865.0111 93.1549 | **20k^+^**  C -0.158185 -0.143486 0.230478  H -0.653371 -0.69509 1.02567  H 0.922924 -0.163847 0.374996  C -0.732447 1.216278 0.020729  H -1.808707 1.337483 0.095066  C 0.020267 2.303595 -0.415006  O -0.585103 3.436268 -0.621745  O -0.567548 -0.708685 -1.028137  H 0.144303 -0.634482 -1.680572  H 1.098155 2.24738 -0.559298  H 0.014224 4.160211 -0.870993  Freq Int  100.8363 14.9396  173.675 6.288  284.8195 3.9751  378.1132 28.2233  383.3674 115.838  549.1357 62.7719  619.3039 146.5589  856.3696 18.7148  944.1015 2.9321  962.7277 50.2921  972.2932 1.7369  1069.9971 13.9169  1157.9182 98.1568  1190.9578 42.4583  1314.9109 64.3771  1334.903 39.9458  1353.24 210.8499  1362.1702 11.7187  1392.8209 2.6172  1509.6698 10.6451  1621.3585 136.6541  3084.4864 8.4929  3149.4828 2.3456  3161.324 0.7874  3190.2841 15.117  3721.6998 462.9394  3765.5059 186.0044 |
| **20l**  C -0.096321 -0.069622 0.179504  H -0.594485 -0.430325 1.091663  H 0.968467 0.068895 0.416045  C -0.690643 1.234157 -0.238064  H -1.760989 1.241411 -0.434135  C 0.032851 2.341294 -0.401866  O -0.418637 3.568907 -0.772966  O -0.270263 -1.019981 -0.879469  H -0.06377 -1.890718 -0.525839  H 1.105005 2.36331 -0.236935  H -1.369256 3.520502 -0.92866  Freq Int  105.1589 0.2896  202.6458 15.7385  259.7747 109.6157  310.6659 22.2826  439.8474 9.362  482.3317 74.5183  557.3555 17.1858  827.8472 11.4425  943.8248 30.6784  972.9166 77.2094  1023.1585 95.5127  1110.1068 92.3021  1145.2582 136.5312  1231.1197 9.2734  1252.6243 78.8794  1289.7893 24.567  1334.7289 10.4435  1396.5337 19.2273  1459.8825 2.978  1512.7118 1.2123  1730.6947 180.4245  2960.7618 73.9294  2990.0374 59.9139  3129.0348 20.62  3177.5053 12.3612  3803.5837 36.2775  3822.9784 11.1045 | **20l^+^**  C -0.010612 -0.081504 -0.00336  H 0.035243 -0.051106 1.109087  H 1.061436 -0.109 -0.304301  C -0.594063 1.180274 -0.478788  H -1.474881 1.116891 -1.115906  C -0.05907 2.421369 -0.141085  O -0.527732 3.566336 -0.528773  O -0.734503 -1.157105 -0.484944  H -0.356476 -1.989662 -0.175999  H 0.818284 2.526575 0.492237  H -1.315666 3.504761 -1.098889  Freq Int  75.4656 33.9032  198.2446 16.2743  200.6876 4.4922  336.3473 94.2596  455.44 2.9592  525.6233 16.0414  579.1024 0.6362  667.4657 125.4011  931.6874 28.1257  1012.592 49.8418  1055.9739 14.6581  1137.9955 203.372  1167.7484 195.4135  1186.7615 1.7784  1214.0903 25.9061  1302.0967 345.6211  1318.4336 8.8939  1354.8195 88.2125  1399.6124 26.7604  1447.5917 77.8825  1615.443 297.006  2832.8556 0.8733  2849.373 111.9753  3151.0502 11.5934  3179.2611 7.6648  3686.1615 274.0013  3810.9419 302.1887 |
| **20m**  C 0.078294 -0.123185 -0.173722  H 0.593627 0.01517 0.787853  H 0.795486 -0.572967 -0.865298  C -0.401879 1.19969 -0.68684  H -0.850096 1.207977 -1.679042  C -0.330076 2.324171 0.028381  O -0.747549 3.563432 -0.34144  O -0.973019 -1.087629 -0.063382  H -1.665494 -0.685395 0.471405  H 0.100038 2.349409 1.024667  H -1.114952 3.520553 -1.232326  Freq Int  113.0193 4.6263  208.5602 11.1528  291.1997 37.3837  371.5805 87.8061  447.7383 29.2513  486.1276 72.7356  559.9337 1.4094  827.9807 14.0506  931.164 38.0227  976.8289 67.7996  1022.4846 140.0876  1105.4823 108.1914  1145.9046 128.4485  1194.5645 8.5615  1269.2509 18.301  1333.4989 16.8634  1351.7598 3.4337  1403.0382 36.098  1431.117 37.5022  1505.6202 1.5563  1719.9878 171.7547  2977.8057 63.0848  3063.3873 35.5031  3124.2298 20.0409  3172.2778 12.3376  3802.472 38.2886  3814.5938 15.5635 | **20m^+^**  C 0.151788 -0.141931 -0.237415  H 0.700957 -0.103022 0.703922  H 0.701413 -0.726414 -0.970696  C -0.281215 1.189438 -0.756031  H -0.605219 1.238032 -1.792429  C -0.43248 2.303445 0.068571  O -0.841974 3.471324 -0.317103  O -1.161493 -0.714047 -0.100359  H -1.486143 -0.607592 0.806491  H -0.17517 2.282638 1.12466  H -1.08608 3.519355 -1.259354  Freq Int  102.5594 11.5723  169.4809 6.2007  286.9152 27.6219  377.0628 33.1406  395.9177 108.141  547.895 15.6967  649.6181 84.1973  845.3555 46.7018  943.9053 13.4964  959.221 49.0501  1011.1662 44.1659  1071.8834 65.7646  1160.0732 79.8012  1185.3038 41.1306  1310.2742 158.7156  1339.1804 27.4603  1358.8899 0.3359  1379.6538 61.9858  1407.6597 5.3538  1508.085 13.7849  1596.1426 273.6999  3089.2416 6.3903  3162.4038 2.7963  3171.2283 2.0123  3179.5253 9.4481  3686.0787 254.0769  3757.9126 204.2415 |
| **21a**  O 1.909390 -0.080053 0.091080  C 0.681002 -0.737562 -0.000520  C -0.432088 0.001317 -0.010522  O -0.377674 1.366386 -0.013570  C -1.821819 -0.538767 -0.000573  H 2.495890 -0.415627 -0.594042  H 0.679366 -1.820075 0.030055  H 0.561772 1.600051 0.007752  H -2.377304 -0.170985 -0.868723  H -1.820145 -1.629515 -0.019820  H -2.355892 -0.204439 0.894386  Freq Int  195.2391 3.6659  244.4526 30.596  270.3697 24.9596  290.4696 92.8808  387.2188 6.8097  535.4703 74.0508  598.5674 78.7816  620.1613 20.9586  741.568 18.3214  909.7933 21.3152  1006.8404 10.0602  1069.0557 6.7021  1110.8361 102.4972  1197.5134 116.5055  1226.6941 87.8639  1370.3387 93.6278  1386.5939 15.5162  1435.1133 5.0648  1467.6561 8.5347  1494.0566 4.527  1760.6829 49.5385  3025.0595 27.1874  3073.2222 19.2221  3116.4702 18.5336  3185.5741 14.3902  3746.1725 50.8743  3817.5268 52.7655 | **21a^+^**  O 1.864275 -0.037987 0.000017  C 0.740314 -0.717241 -0.000018  C -0.456823 0.036289 -0.000010  O -0.407394 1.337702 -0.000007  C -1.799515 -0.578260 0.000006  H 2.662065 -0.590854 -0.000002  H 0.741521 -1.801143 -0.000028  H 0.503660 1.685342 0.000009  H -2.356610 -0.234764 -0.878716  H -1.753107 -1.665940 -0.000190  H -2.356426 -0.235095 0.878980  Freq Int  84.4284 2.316  217.5168 13.7924  267.2311 1.618  410.1984 5.3145  474.4415 124.0682  594.9791 16.5774  619.94 19.1739  674.3644 151.1438  822.4131 3.3349  899.6352 35.7418  1022.1273 6.1085  1025.18 4.7842  1147.7715 43.3119  1220.79 137.6201  1314.8449 247.4272  1387.0897 175.2184  1393.4083 17.9345  1446.5093 18.8336  1479.5438 13.3563  1506.007 53.5339  1636.0049 13.1408  3027.641 25.5468  3078.3497 5.5712  3150.3234 1.9245  3205.4003 17.6593  3670.3605 143.2683  3747.8217 386.766 |
| **21b**  O -1.922825 0.139057 -0.071871  C -0.786105 -0.639951 0.031725  C 0.431247 -0.096365 0.020916  O 1.517376 -0.950658 -0.072185  C 0.713993 1.371936 0.004114  H -2.641052 -0.310812 0.380656  H -0.909562 -1.718443 0.035654  H 2.258461 -0.547787 0.391296  H 1.204693 1.664761 -0.930732  H -0.208971 1.941032 0.107500  H 1.385211 1.650334 0.827544  Freq Int  173.9049 82.3112  193.2105 35.3107  252.4711 58.2644  284.5208 95.5859  326.5531 27.3606  421.6489 6.7651  454.0525 13.3785  592.385 3.2149  831.7869 29.7484  833.5961 14.2494  1016.5108 3.6086  1061.1618 3.8696  1131.6652 248.1729  1192.3922 231.1403  1257.9723 10.7473  1315.213 1.464  1384.1735 11.8629  1423.2605 26.5553  1481.1618 6.8758  1488.225 8.7515  1789.9502 2.7048  2997.3641 46.2104  3045.4733 28.7551  3132.5177 5.9468  3159.7963 16.7189  3819.7616 36.1892  3850.2377 69.4759 | **21b^+^**  O -1.852527 0.204959 0.000000  C -0.841649 -0.622323 0.000000  C 0.463126 -0.090297 0.000001  O 1.396669 -1.011434 0.000000  C 0.782245 1.356277 0.000000  H -2.711685 -0.246979 0.000000  H -0.996971 -1.697481 -0.000001  H 2.295223 -0.642852 0.000001  H 1.378820 1.606368 -0.885179  H -0.119719 1.964442 0.000027  H 1.378869 1.606359 0.885149  Freq Int  63.5323 0.8222  235.8191 13.8198  270.4715 5.1322  446.5648 10.2706  457.2988 69.0151  571.4534 212.9032  596.1635 3.2679  615.1939 6.9271  832.6083 17.015  885.0387 0.4047  1023.6447 1.4318  1026.0159 10.6791  1148.057 70.0777  1228.6768 255.8215  1329.2579 211.4875  1395.4691 36.577  1418.2202 58.9933  1463.6745 68.6284  1465.2075 16.3762  1486.3199 44.9921  1662.6828 1.6238  3020.7978 13.1604  3072.995 2.0086  3158.5415 2.9023  3180.5311 17.573  3733.4165 435.3762  3746.3321 193.351 |
| **21c**  O 1.971720 -0.179030 -0.010461  C 0.725856 -0.733670 -0.010884  C -0.405595 -0.025505 -0.001839  O -0.268935 1.363119 0.080655  C -1.791271 -0.580602 0.004957  H 1.844356 0.779864 -0.009525  H 0.738983 -1.814622 0.001626  H -0.900085 1.776356 -0.518164  H -2.366997 -0.236279 -0.865070  H -1.774319 -1.671676 -0.025306  H -2.338159 -0.267694 0.901484  Freq Int  210.0263 2.1261  238.7963 18.8391  269.3496 40.0187  280.9558 77.112  378.5818 3.644  502.1682 36.0448  550.1466 88.2171  614.9793 4.554  841.342 36.0671  874.9332 49.7048  1002.8015 8.3951  1063.0493 3.2985  1150.0787 222.7682  1203.9161 4.2783  1264.3968 132.9396  1317.4575 42.9933  1411.0126 38.6929  1423.6764 21.0482  1470.9807 9.6491  1495.631 12.3037  1774.4387 42.7415  2990.4948 54.5353  3037.7562 31.7086  3104.7547 17.2005  3218.3577 8.8186  3763.32 49.3367  3807.6487 32.8857 | **21c^+^**  O 1.942739 -0.171209 -0.000002  C 0.768517 -0.722090 0.000001  C -0.435146 0.016521 -0.000003  O -0.254065 1.321950 0.000007  C -1.782011 -0.595924 0.000000  H 1.903152 0.803976 -0.000002  H 0.773252 -1.805295 0.000005  H -1.077749 1.835846 -0.000032  H -2.344170 -0.273867 -0.884972  H -1.727888 -1.683747 -0.000013  H -2.344151 -0.273886 0.884991  Freq Int  105.0473 0.6101  217.6725 6.2936  265.485 4.5385  399.4867 1.744  493.5787 30.4863  551.2414 85.3538  610.8664 13.7797  674.3299 108.2005  903.375 54.494  927.554 50.762  1012.0545 1.088  1022.7737 19.4647  1137.9283 110.7202  1236.9618 145.3191  1367.4946 249.6524  1384.3247 42.0399  1404.1055 27.2091  1455.7624 17.1208  1457.7166 90.6403  1489.8579 24.4078  1646.5254 16.1199  3014.9162 15.244  3066.2759 2.5812  3142.9405 0.7269  3221.6197 19.7516  3663.063 160.4141  3740.0783 266.2863 |
| **21d**  O -1.964465 0.038837 -0.029019  C -0.767010 -0.637074 0.008351  C 0.405503 0.001389 0.010026  O 0.431993 1.378678 -0.024431  C 1.724577 -0.706465 -0.007611  H -2.667662 -0.574814 0.194713  H -0.798123 -1.720314 -0.013445  H 1.316158 1.673539 0.210851  H 2.293778 -0.460198 -0.912311  H 2.338978 -0.415941 0.855154  H 1.598226 -1.789484 0.028036  Freq Int  86.8899 49.8394  152.3975 160.4232  216.3511 26.3573  246.5709 2.6086  292.5686 6.5044  389.3932 2.701  550.6813 7.0697  602.9409 18.2005  770.2937 37.8816  875.1557 43.5196  1003.414 5.6852  1065.674 3.8325  1149.323 124.9389  1201.1064 28.0617  1244.2518 271.3671  1340.6351 99.9704  1403.0412 13.48  1422.4283 12.6083  1471.6892 8.535  1496.8624 4.5622  1803.6146 13.9126  2991.6146 55.6849  3030.7199 34.1031  3108.6925 16.8825  3172.7731 17.6261  3843.6763 52.235  3868.721 73.8897 | **21d^+^**  O -1.898384 0.022745 0.000041  C -0.791234 -0.663964 0.000021  C 0.439447 0.029288 0.000016  O 0.350909 1.332766 0.000051  C 1.748223 -0.668880 -0.000006  H -2.692325 -0.535895 -0.000443  H -0.812801 -1.748549 -0.000007  H 1.216554 1.773000 -0.000441  H 2.327947 -0.379785 -0.884630  H 2.327988 -0.379668 0.884549  H 1.633823 -1.751857 0.000051  Freq Int  100.7275 0.6697  208.8697 2.1578  272.4321 4.0394  407.7114 2.9763  481.6415 75.3708  585.0483 209.1098  605.8602 14.1414  633.4914 4.1186  865.9184 0.0647  891.8959 45.1982  1016.3061 3.1976  1027.3297 13.8669  1149.9802 129.5852  1237.8975 73.873  1335.5593 236.7037  1374.6119 102.7545  1401.0571 24.6665  1459.3334 16.0395  1467.9814 77.3134  1506.7632 50.2868  1668.87 0.3701  3018.3147 12.8438  3070.1586 2.1044  3143.3793 0.4309  3193.2349 13.8409  3732.0167 417.0537  3745.191 187.5816 |
| **21e**  O 1.925475 0.101270 0.096698  C 0.764848 -0.650102 -0.007729  C -0.442733 -0.077740 -0.012951  O -1.610376 -0.804194 0.001840  C -0.694200 1.392685 -0.012124  H 2.534155 -0.178837 -0.594070  H 0.882367 -1.733664 0.004440  H -1.399311 -1.744170 0.028817  H -1.219732 1.688628 0.901213  H 0.246126 1.937380 -0.073061  H -1.331886 1.664996 -0.858819  Freq Int  161.6871 9.6328  240.911 139.3476  268.3493 22.4572  287.2987 12.66  386.2976 107.511  428.5995 17.0537  475.3835 2.8675  588.7677 3.5231  781.154 42.0371  845.645 5.3567  1017.5942 24.246  1070.433 0.0514  1137.4175 63.4556  1197.5425 366.4219  1248.8657 7.7096  1311.4524 5.5273  1387.8605 16.016  1446.022 10.0875  1480.4442 8.1754  1486.4126 3.8844  1758.1135 17.9867  3027.7385 27.5565  3073.8098 20.4239  3101.3141 40.0531  3144.5593 6.7306  3810.3311 22.7367  3812.0568 43.7378 | **21e^+^**  O 1.846915 0.187101 -0.000002  C 0.826079 -0.630796 0.000001  C -0.473595 -0.078374 -0.000008  O -1.521405 -0.861130 -0.000003  C -0.755111 1.371901 -0.000004  H 2.703524 -0.269860 0.000004  H 0.994446 -1.705657 0.000010  H -1.323264 -1.812439 0.000022  H -1.359576 1.620178 0.879274  H 0.156689 1.963532 -0.000299  H -1.360137 1.620090 -0.878913  Freq Int  20.5672 3.3488  220.4778 18.1913  278.206 1.4454  458.031 47.1629  458.0421 16.2803  573.3046 5.0857  587.6614 4.7972  610.9552 225.3243  837.1025 10.8177  850.0366 6.2518  1022.1354 4.1324  1033.3936 2.701  1149.0138 198.7818  1233.1252 13.3862  1329.4529 297.3932  1389.345 61.2255  1402.1665 130.1046  1453.1952 19.0932  1465.6018 14.4191  1497.1884 21.5651  1642.2483 8.1437  3031.2364 23.0907  3081.298 4.7608  3153.7842 12.7156  3170.4251 2.6954  3718.4318 166.6352  3744.1226 344.9641 |
| **21f**  O 2.009312 -0.009854 0.001336  C 0.797169 -0.646390 0.028692  C -0.416914 -0.090891 0.011230  O -1.509646 -0.945569 -0.092950  C -0.734001 1.373884 -0.006581  H 1.873649 0.941205 -0.059900  H 0.904252 -1.722897 0.042201  H -2.160655 -0.690507 0.570201  H -1.392413 1.642136 0.829767  H 0.154401 2.005526 0.080974  H -1.254086 1.648298 -0.930381  Freq Int  136.7329 11.3018  247.2236 89.6184  270.1555 7.5422  284.484 69.1178  361.3787 26.0153  425.5825 10.971  452.8861 48.7269  582.9772 7.0075  818.6992 1.0328  880.6161 33.0379  1012.3298 27.3719  1049.0712 5.4878  1139.2001 328.8408  1190.6035 75.7896  1277.1658 11.1091  1315.3704 49.9099  1388.9035 9.7992  1424.7606 12.7574  1470.9793 9.3061  1491.4503 4.6782  1767.8058 26.1899  2999.6002 35.2381  3047.8352 27.0177  3081.1105 33.8382  3211.2981 6.5492  3790.9907 21.8057  3826.9435 20.6929 | **21f^+^**  O 1.961441 0.061924 -0.000003  C 0.855853 -0.620649 0.000004  C -0.450849 -0.089295 0.000006  O -1.374681 -1.023037 -0.000004  C -0.816218 1.350646 0.000001  H 1.845091 1.026576 -0.000012  H 0.996676 -1.696564 0.000010  H -2.280141 -0.671946 -0.000001  H -1.417471 1.584276 0.886036  H 0.046177 2.017998 0.000202  H -1.417129 1.584353 -0.886247  Freq Int  13.3804 0.3363  216.5509 2.6934  271.2782 3.0467  434.0749 9.1046  447.5537 3.7771  553.161 67.8593  584.798 10.1178  623.7649 146.0566  820.6783 5.0425  940.6149 41.7987  1018.8464 13.3704  1019.0411 8.936  1142.684 359.5247  1210.6345 33.2584  1376.9554 1.7983  1396.7865 94.3678  1416.0458 84.3654  1446.4894 122.9113  1454.0102 17.7373  1492.9241 34.9436  1647.372 32.3639  3022.3356 11.8415  3078.0795 3.5124  3122.9676 1.8411  3202.3977 24.0416  3718.9459 170.607  3736.2043 214.1045 |
| **21g**  O 2.006604 -0.012946 -0.000015  C 0.784507 -0.645364 0.000022  C -0.427130 -0.080968 0.000011  O -1.593947 -0.823690 0.000006  C -0.730508 1.382810 -0.000005  H 1.872460 0.939973 -0.000031  H 0.909201 -1.722949 0.000063  H -1.372229 -1.760961 -0.000152  H -1.320781 1.648287 -0.882449  H -1.320871 1.648235 0.882396  H 0.169752 2.001628 0.000084  Freq Int  63.5714 19.6618  203.5019 46.6212  275.7463 11.1095  285.3858 33.3932  365.3857 87.8922  427.3395 23.846  447.7489 22.7533  580.3231 4.0564  821.4941 44.2562  829.2325 1.323  1009.9334 57.7894  1060.7464 0.0127  1130.1929 113.8952  1210.9236 199.2927  1234.1483 104.7419  1341.4598 13.1438  1412.7468 50.4931  1445.8719 10.7988  1463.856 9.8674  1496.1008 4.042  1759.5561 0.5214  3022.9051 23.2973  3073.8545 17.7191  3095.4785 32.0634  3171.1387 18.9022  3821.6427 29.4876  3833.0863 18.5517 | **21g^+^**  O 1.936614 0.007483 -0.004041  C 0.817014 -0.657884 0.005630  C -0.469968 -0.077515 0.007837  O -1.535124 -0.836437 -0.002136  C -0.723883 1.385014 0.002467  H 1.832979 0.974305 -0.011828  H 0.960978 -1.734991 0.011691  H -1.355817 -1.792343 -0.021976  H -0.567566 1.790791 -1.005275  H -1.757518 1.579491 0.286717  H -0.063948 1.916687 0.694485  Freq Int  37.0605 1.0309  216.6255 1.9487  256.1999 11.7186  431.4312 15.781  464.1637 19.9657  578.282 8.8292  588.8851 208.0129  624.5892 2.6514  830.2534 8.2638  905.3207 72.5701  1018.5787 24.6751  1029.6178 3.2662  1160.1616 19.7324  1214.8906 277.6248  1375.1519 167.7009  1397.2805 91.824  1405.7099 81.5715  1450.3093 17.7722  1460.1338 23.6225  1492.4856 27.942  1632.1819 1.2258  3017.9618 16.2324  3080.4761 1.9344  3142.7271 5.133  3177.7881 15.4094  3705.4422 285.0379  3713.9338 23.2645 |
| **24**  C 1.718551 -0.860709 -0.340605  H 1.268736 -1.483277 -1.117814  H 2.026708 -1.524219 0.474618  H 2.602414 -0.353008 -0.725299  C 0.739214 0.152435 0.200916  C -0.720355 -0.260794 0.325794  H -0.771953 -1.206729 0.884329  O -1.488690 0.729922 0.949214  O 1.069452 1.266513 0.548123  H -0.885579 1.480674 1.069624  H -1.090689 -0.475608 -0.689850  Freq Int  77.5272 0.209  120.1923 5.3875  280.3476 12.4304  379.4438 52.1244  409.7388 11.5935  482.5066 34.9401  619.7876 9.38  806.3389 11.7066  859.9859 3.8265  977.2751 16.3753  1095.271 3.1918  1118.313 137.3798  1202.043 22.1767  1247.059 2.548  1308.478 69.2886  1389.867 46.2464  1445.779 83.9453  1465.788 4.9103  1477.244 9.7572  1487.225 13.7613  1788.172 148.2129  2962.101 38.3132  2994.891 30.4615  3032.959 2.758  3093.437 9.7922  3139.577 7.408  3691.042 71.4914 | **TS1**  C 1.743276 -0.768183 -0.391441  H 1.236467 -1.601015 -0.879788  H 2.442468 -1.161662 0.354611  H 2.326992 -0.198312 -1.117768  C 0.753663 0.090784 0.306708  C -0.648961 -0.109096 0.519065  H -0.833070 -0.300300 1.581175  O -1.395018 -0.996738 -0.274282  O 1.064518 1.219066 0.832869  H -0.216707 1.289330 0.888700  H -1.692378 -0.513143 -1.049318  Freq Int  -2133.26 357.0184  71.4433 1.8023  222.808 3.325  276.6122 57.8558  306.7981 33.0332  434.3107 17.7991  518.0375 11.0084  632.3959 8.6635  708.8834 132.8851  840.8328 8.9815  1019.593 7.7382  1036.476 29.7641  1108.196 214.1977  1168.575 72.156  1260.968 15.053  1346.178 56.8853  1386.009 40.5319  1426.179 17.8987  1464.718 60.3098  1484.624 37.0654  1524.459 87.5641  1887.019 15.353  3031.868 2.2451  3058.914 10.5869  3092.9 7.3689  3130.606 5.199  3841.836 49.2563 |
| **TS2**  C 1.439513 -0.626258 -0.264657  H 1.097779 -1.537632 -0.757326  H 2.068206 -0.92128 0 0.591967  H 2.071220 -0.077126 -0.967132  C 0.280670 0.247826 0.200362  C -0.923490 -0.402969 0.600912  H -1.356232 -0.512825 1.597906  O -1.570881 -0.815527 -0.425436  O 0.670660 1.355282 0.963462  H 1.550437 1.195069 1.327506  H -0.669363 -0.07563 0 -0.966524  Freq Int  -2262.66 289.304  232.0026 5.8211  278.0589 1.0957  291.3439 76.1645  337.3899 76.7557  385.0196 25.2295  407.5954 0.578  661.7424 4.0965  825.2444 62.1542  947.8408 7.4741  1001.132 37.7467  1022.913 25.6418  1102.8 15.429  1177.662 41.0999  1186.877 85.5014  1271.998 143.2713  1400.578 1.8335  1408.679 45.6665  1475.515 10.1994  1497.179 55.6811  1549.413 114.1508  1843.844 11.4873  2945.553 86.2125  3062.846 21.6055  3107.87 25.7262  3115.619 11.3544  3752.501 7.8241 | **TS3**  C 1.646593 -0.824117 -0.186794  H 1.256408 -1.753713 -0.604813  H 2.297575 -1.065774 0.659329  H 2.252953 -0.311156 -0.936020  C 0.542779 0.039965 0.301140  C -0.873594 -0.271534 0.436786  H -1.176308 -1.328088 0.388559  O -1.570760 0.636377 1.001589  O 0.783269 1.258108 0.688064  H -0.154901 1.501900 1.049191  H -0.581836 -0.077649 -0.803371  Freq Int  -878.959 252.7713  207.0076 0.3312  299.9316 2.611  318.5438 47.9189  387.2831 68.4985  589.2711 8.1765  661.128 8.4019  943.4615 28.0959  1000.314 4.7331  1025.409 16.9367  1037.454 56.8517  1124.941 26.5381  1185.559 8.0213  1250.931 47.1727  1281.533 29.0302  1405.264 0.7618  1453.349 107.3803  1467.182 36.3286  1475.531 115.8166  1502.909 111.5083  1562.764 191.9324  1811.709 86.6354  2736.196 214.8158  3001.813 46.3852  3035.299 9.0713  3095.632 3.816  3123.066 8.7636 |
